# The Results of a 12-Month Open-Label Follow-Up Study with MRI Monitoring of Patients with Parkinson’s Disease After MRI-Guided FUS

**DOI:** 10.3390/jcm14238329

**Published:** 2025-11-24

**Authors:** Elena Anatolievna Katunina, Mikhail Yurievich Martynov, Vsevolod Vadimovich Belousov, Nataliya Vladimirovna Titova, Mikhail Borisovich Dolgushin, Raisa Tairovna Tairova, Natalia Nikolaevna Shipilova, Madina Zamirovna Ivanova, Ilya Vladimirovich Senko, Ivan Sergeevich Gumin, Vijay Mais-ogly Dzhafarov

**Affiliations:** 1 Federal Center of Brain Research and Neurotechnologies, Russian Federal Medical and Biological Agency, Ostrovityanova Str., 1, Building 10, 117513 Moscow, Russia; elkatunina@mail.ru (E.A.K.); m-martin@inbox.ru (M.Y.M.); belousov@fccps.ru (V.V.B.); dolgushin.m@fccps.ru (M.B.D.); tairova-r@mail.ru (R.T.T.); natali.33@mail.ru (N.N.S.); ivanovamadina97@gmail.com (M.Z.I.); senko@fccps.ru (I.V.S.); gumin.i@fccps.ru (I.S.G.); djafarov.v@fccps.ru (V.M.-o.D.); 2 Institute of Neurosciences and Neurotechnologies, N.I. Pirogov Russian National Research Medical University, Ministry of Health of the Russian Federation, Ostrovityanova Str., 1, Building 6, 117513 Moscow, Russia

**Keywords:** Parkinson’s disease, tremor, magnetic resonance guided focused ultrasound (MRgFUS), FUS thalamotomy, quality of life (QoL), activities of daily living (ADL), anxiety, depression, dementia, mild cognitive impairment (MCI)

## Abstract

**Background:** Tremor-dominant Parkinson’s disease (TDPD) is the most common subtype of PD. Tremor is difficult to treat and less than 50% of patients respond to dopaminergic medications. Magnetic resonance guided focused ultrasound (MRgFUS) thalamotomy is an incisionless noninvasive method for treating pharmacoresistant tremor in PD patients, but its effect on progression of PD is unknown. In this study, we investigate the efficacy of MRgFUS thalamotomy on progression of motor and non-motor symptoms, using a levodopa equivalent daily dose (LEDD) requirement. **Methods:** A total of 21 PD patients with ineffective tremor correction by medical therapy underwent MRgFUS thalamotomy. Assessments of motor and non-motor symptoms, adverse events (AE), changes in LEDD, and evolution of FUS (focused ultrasound) lesion were performed on the day before surgery, and then 2 days, as well as 3, 6, and 12 months, after the procedure. **Results:** On the 2nd day after FUS thalamotomy, 11 patients were tremor-free and, in 10 patients, tremor decreased by 80–90% with a concomitant reduction in hypokinesia and rigidity. By the end of the 12th month, 5 patients remained tremor-free; in 11 patients, mild/moderate tremor re-emerged; and in 5 patients, there was a relapse of severe tremor. Quality of life (QoL) and activities of daily living (ADL) improved significantly at 3 months and remained stable thereafter. Cognitive function improved in patients with baseline MoCA score < 26 points at 3 months after FUS. Anxiety progressed between baseline and end of follow-up. By the end of the follow-up period, LEDD was lowered or stable in 9 patients. Four patients had persistent mild AE. **Conclusions:** This open label study suggests a beneficial effect of MRgFUS in reducing tremor, hypokinesia, and rigidity and improving QoL, ADL, and cognitive function in TDPD patients in the short term, although long-term data needs to be collected in further studies.

## 1. Introduction

Rest tremor is one of the most common symptoms of Parkinson’s disease (PD). According to the dominant motor symptom, PD is classified into tremor-dominant (TDPD), intermediate, and postural instability/gait difficulty subtypes [1,2]. In the DATATOP study, 55% of patients had the tremor-dominant subtype, while the indeterminate and postural instability/gait difficulty subtypes were found in 16% and 29% of patients [3]. Tremor-dominant PD is characterized by a slower progression [4], a lower risk of dyskinesias, and salient non-motor symptoms, including cognitive disorders, hallucinations, and psychosis [1,5].

For most patients with PD, dopaminergic therapy remains the first-line treatment for correction of akinesia and rigidity, but it is less effective in alleviating tremor [6,7]. Zach et al. demonstrated that tremor responded to dopaminergic medications in 28% of patients (DOPA-sensitive subgroup), while in 33% and in 39% of patients, tremor diminished minimally or was DOPA-resistant [6]. To treat resistant tremor, higher doses of levodopa or dopamine receptor agonists are administered, which in turn increases the risk of adverse events (AE) and drug-induced dyskinesias.

Another approach is to treat resistant tremor via surgical intervention such as DBS or radiofrequency ablation. Previous studies have shown that selective high-frequency stimulation of the thalamic ventral intermediate (VIM) nucleus suppressed tremor in patients with essential tremor (ET) or PD [8,9]. Recently, several research teams have demonstrated immediate and long-term beneficial effects of magnetic resonance guided focused ultrasound (MRgFUS) for correction of tremor, quality of life (QoL), and memory functions in patients with ET [10,11,12].

For alleviating motor symptoms in patients with PD, several targets including the VIM, subthalamic nucleus, and internal segment of the globus pallidus were investigated [13,14,15,16]. To achieve accurate VIM targeting during FUS thalamotomy, anatomical landmarks or neural tracks created on diffusion tensor MRI were used [17]. Besides targeted destruction of the VIM, local thermal ablation induced edema and disruption of neural tracks, which may affect long-term clinical outcomes. The association between temporal evolution of edema, fractional anisotropy, tremor improvement, and potential side effects is still not fully understood [18].

In 2018, the FDA approved MRgFUS thalamotomy for treating refractory tremor in patients with TDPD. To date, the results of published trials, including randomized clinical trials, suggest that in TDPD patients, MRgFUS thalamotomy reduces tremor, improves QoL, has a beneficial effect on cognitive functions, and is safe [19,20,21,22]. However, the number of long-term observational studies is limited and there is a scarcity of data assessing the impact of MRgFUS on non-motor symptoms, including QoL, activities of daily living (ADL), anxiety, depression, cognitive function, changes in levodopa equivalent daily dose (LEDD), and MRI (magnetic resonance imaging) evolution of post-FUS (focused ultrasound) changes in the lesion area.

The aim of this study was to prospectively evaluate the immediate and 3-, 6-, and 12-month effect of MRgFUS on tremor, other motor and non-motor symptoms, LEDD, and MRI evolution of post-MRgFUS lesion.

## 2. Materials and Methods

**Figure 1:**
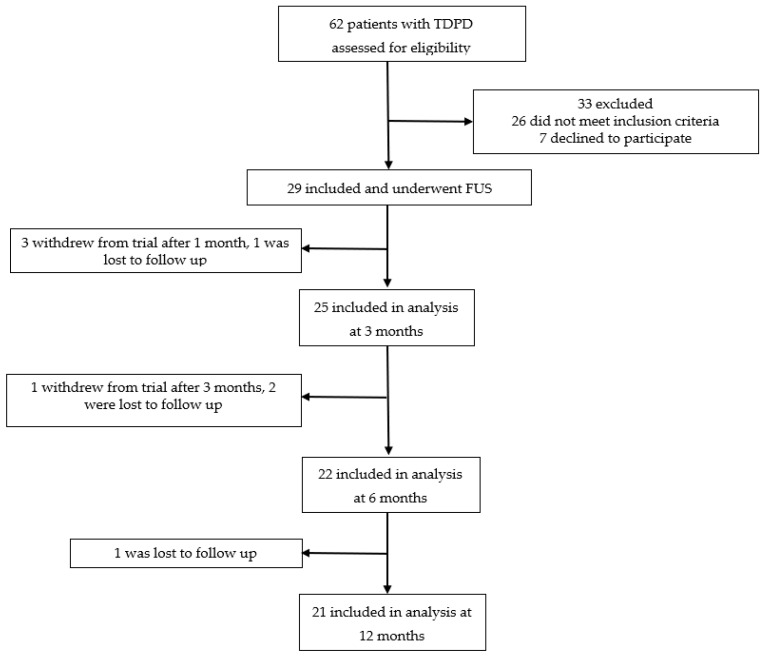
Flowchart of the patient selection in the study.

This was a single-center prospective study. From December 2021 to June 2023, we screened 62 patients with TDPD and 29 were included and underwent MRgFUS thalamotomy. During the follow-up, 8 patients were lost or withdrew consent and 21 patients were included in the final analysis (Figure 1).

The study was conducted in accordance with the 1964 Helsinki Declaration and approved by the ethical committee of the Federal Center of Brain Research and Neurotechnologies (protocol code 17/06-06-21, date of approval 6 June 2021). All patients signed an informed consent form before enrollment in the study. Prior to MRgFUS, patients were counseled about the surgical technique and possible side effects. They were informed that the procedure requires shaving the entire head and necessitates a skull CT to determine skull bone density.

The diagnosis of the PD was made according to MDS Clinical Diagnostic Criteria for PD [23] by a movement disorder neurologist. The procedure was funded for all patients through state budgetary allocations as part of a government-guaranteed high-technology healthcare program.

### 2.1. Inclusion/Exclusion Criteria

The inclusion criteria were (1) TDPD with ineffective tremor correction by medical therapy for at least 1 year; (2) postural or action tremor ≥ 2 points on the Clinical Rating Scale for Tremor (CRST) [24]; (3) age > 40 and < 75 years; (4) MoCA > 21; (5) skull density ratio ≥ 0.4; and (6) ability and willingness to give informed consent and to attend all study visits.

Patients were excluded if they had (1) neurodegenerative disease/syndrome other than PD; (2) motor fluctuations and/or levodopa-induced dyskinesias; (3) individual intolerance or refusal to take levodopa or other dopaminergic medications; (4) previous DBS (deep brain stimulation) or stereotactic brain ablation; (5) moderate or severe depression (Beck Depression Inventory ≥ 19); (6) psychiatric disease; (7) previous stroke, transient ischemic attack, aneurism, or arteriovenous fistula; (8) seizures within the past year; (9) coagulopathy or abnormal bleeding; (10) uncontrolled hypertension; (11) myocardial infarction within the past 6 months, unstable angina pectoris on medications, or congestive heart failure requiring medications other than diuretics; (12) contraindications for high-field MRI; (13) pregnancy or lactation; or (14) claustrophobia.

### 2.2. Assessments

All patients underwent motor and non-motor assessments in the morning 1 h after administration of antiparkinsonian medications (“ON” medication state). The assessments were made on the day before FUS thalamotomy, on the 2nd day after, and at the end of the 3rd, 6th, and 12th months.

To assess motor symptoms, we used CRST, part III of the Unified Parkinson’s Disease Rating Scale (UPDRS) [25], and the Tinetti Balance and Mobility Scale [26]. Changes in the treated hemibody were evaluated with hemi-CRST (items 5–6, 8–9, 11–14) and hemi-UPDRS (items 20–26).

To assess non-motor symptoms, the following scales were used: the Non-Motor Symptoms Scale for PD (NMSS) [27], Montreal Cognitive Assessment (MoCA) [28], Beck Depression [29] and Anxiety Inventory [30], and Apathy Scale [31]. To prevent patient training effects during repeated cognitive testing, we used different versions of the MoCA test for each session. For assessment of impulsive–compulsive disorders, patients were subjected to the Questionnaire for Impulsive–Compulsive Disorders (ICD) in the Parkinson’s Disease Rating Scale—QUIP-Short (presence of at least 1 positive answer for each item) and QUIP-Full (presence of 2 or more positive answers for pathological gambling or binge eating, 1 for hypersexuality, oniomania, ICD-associated disorders, and positive answers to at least questions 1 and 4 for establishing dopamine dysregulation syndrome (DDS)) [32].

ADL and QoL were evaluated with the Schwab and England ADL Scale (S&E ADL) [33] and Parkinson’s Disease Quality of Life Questionnaire-39 (PDQ-39) [34], respectively.

Besides motor and non-motor symptoms, we also assessed changes in LEDD between the baseline and 3, 6, and 12 months after MRgFUS [35].

Adverse effects (AE) were divided as thalamotomy- (owing to ablation of VIM) or procedure (due to FUS or MRI)-related and were documented at all study follow-up points. We used Clavien–Dindo criteria to classify the severity of AE [36].

### 2.3. MRI

**Figure 2:**
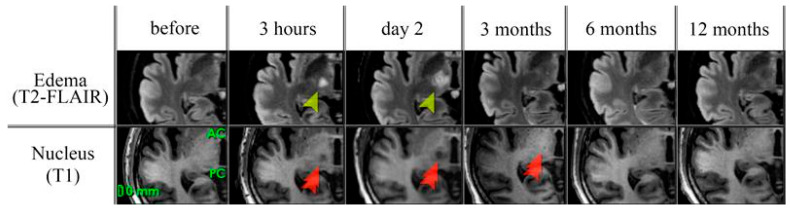
MRI follow-up before and after MRgFUS. Edema (yellow arowhead) was assessed on T2-FLAIR images, the nucleus of destruction (red double arowhead) was assessed on T1 images. The changes within VIM area were visible only at 3 h and 2 days after MRgFUS.

All patients underwent an MRI scan of the brain using a Discovery MR750w 3.0 T MRI scanner (GE Healthcare, Chicago, IL, USA) with a neurovascular coil. The MRI was performed on the day before MRgFUS, 3 h after surgery, on the 2nd day after, and 3, 6, and 12 months after MRgFUS. The MRI protocol included T1-weighted images (T1-WI) with an isotropic voxel of 1 mm, T2-FLAIR images with an isotropic voxel of 1.2 mm, susceptibility-weighted images (SWI) with an axial size of voxel 0.4 × 0.4 mm and thickness 1.6 mm, diffusion-weighted images (DWI) with an axial size of voxel 1 × 1 mm and thickness of 3 mm, diffusion-tractography images (DTI) with an isotropic voxel of 2.5 mm, and 64 directions. The whole examination took about 45 min. The nucleus (lesion area) of MRgFUS destruction was defined as the area of hypointensity on T1-WI images and edema as the area of hyperintensity on T2-FLAIR images (Figure 2). Both the nucleus and edema were identified manually on each section of the T1-WI and T2-FLAIR series. To assess structural integrity of the white matter before and after MRgFUS, fractional anisotropy (FA) maps were generated using scanner’s built-in software (version DV25, GE Healthcare, Chicago, IL, USA). The images were combined with the FA maps using automatic co-registration to obtain mean FA values in the marked areas [37].

**Figure 3:**
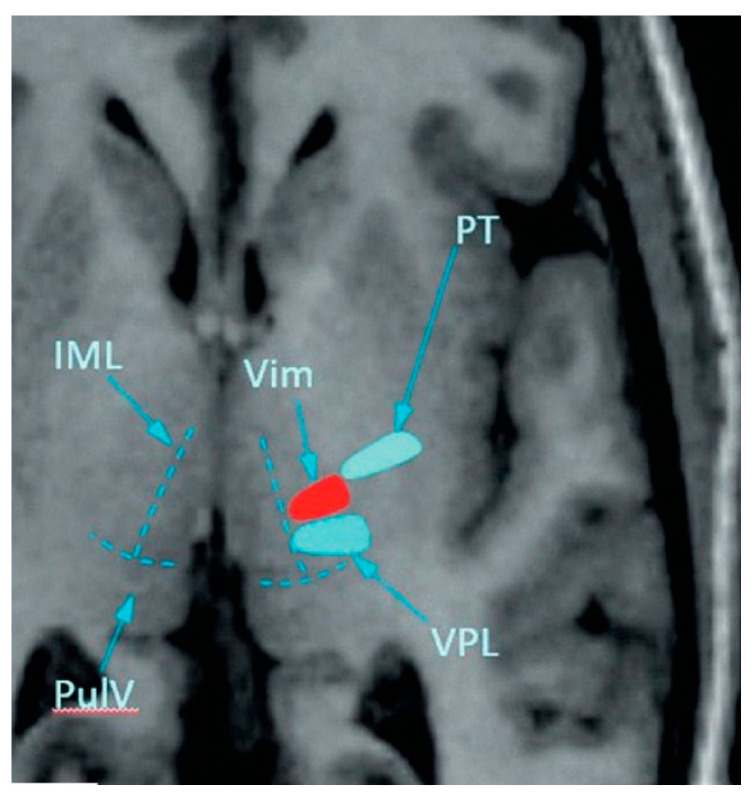
VIM targeting during FUS thalamotomy. VIM—ventral intermediate nucleus; VPL—ventral posterolateral nucleus; IML—internal medullary lamina (thin Y-shaped layer of myelinated nerve fibers delimiting anterior, medial, and lateral groups of thalamic nuclei); PuLV (pulvinar thalami)—pulvinar nuclei; PT—posterior limb of the internal capsule (adapted from Dolgushin MB. et al.) [40].

Since all patients had bilateral symptoms, the thalamus to be treated was chosen contralateral to the side with greater tremor. Initial VIM targeting was performed with built-in software as described by Benabid AL. et al. [38] at 25% of the AC-PC distance anterior to the PC, 14 mm lateral to the AC-PC line, and 1–2 mm above the intercommissural plane (Figure 3). Minor corrections to the initial target’s location (particularly in patients with enlargement of the 3rd ventricle) were made to adjust for individual patient anatomy [19,39]. Starting with the 11th patient, we combined traditional anatomical targeting with MR tractography-guided targeting.

### 2.4. MRgFUS Treatment

On the day of the surgery, all patients were in the “ON” medication state. For MRgFUS thalamotomy we used a specialized platform with a FUS transducer (Exablate Neuro, Insightec, Haifa, Israel) connected to the MRI scanner. The protocol included (1) calibration sonication to assess stereotactic accuracy without inducing any clinical effects; (2) test sonication; and (3) therapeutic sonications to achieve clinical effect [39].

At each stage, sonication temperature range and power range were adjusted as described by Zaaroor M. et al. [39]. The sonication temperature range was 40–46° C at the calibration stage and increased to 46–52 °C to achieve temporary tremor reduction at the test sonication stage. After sufficient tremor reduction was achieved and AE were absent, the temperature was increased to 57–62 °C to achieve lasting tremor reduction. The sonication pulse power increased at every stage from 450 to 500 W at the calibration stage to 700–800 W at the ablation stage. The number of sonications varied from 6 to 17 (mean—9.9 ± 2.5). The bone density coefficient was 0.58 ± 0.1 (range 0.4–0.85).

If there was insufficient tremor reduction or side effects such as paresthesias or mild paresis during the test sonication, the focal point’s location was adjusted until tremor suppression without side effects was achieved. Once the optimal location was identified, the temperature was increased, and tissue destruction of the target area was performed.

### 2.5. Sample Size Calculation and Statistical Analysis

To calculate sample size, we used the preliminary results of 12 months’ monitoring of 7 patients with TDPD and FUS ablation of VIM in our center. As the primary end point, we assessed changes in hemi-CRST a,b on the treated side. In this cohort, the effect size for repeated measures (Cohen’ d*_RM_*) was 1.154 and correlation in hemi-CRST a,b between the baseline and end of the 12th month was r = 0.489. Standard deviations (SDs) at baseline and at 12 months’ follow-up were 5.6 and 6.9, respectively. While determining sample size, we also analyzed the results from previous studies by Bond AE. et al. [19] and by Yamamoto K. et al. [21].

For the present study, statistical power was set at 80% with α = 0.05. Effect size (Cohen’s d*_RM_*) was set at 0.9. We assumed that SDs would not differ from SDs in our exploratory study of 7 patients. The sample size was estimated to be 21 patients. Since most of the patients were from various parts of the country, we set a drop-out rate ≈ 50%. Altogether, 29 patients were included in the study and 8 patients dropped out during the follow-up.

The statistical analysis was performed using SPSS software, version 23.0 (SPSS Statistics, IBM Corporation, Armonk, NY, USA). The normality of data was analyzed by Shapiro–Wilk test. Depending on normality of distribution, demographic variables, scores of motor and non-motor tests, volumes of nucleus of destruction and edema, and FA values were presented as mean and standard deviation [SD] or median and interquartile range [IQR]. To examine differences between related variables, we used paired-samples *t*-tests, Wilcoxon signed-rank tests, or Friedman’s Two-Way Analysis of Variance by Ranks. If the difference between variables was significant, to assess the true change, we calculated the minimal detectable change (MDC). The MDC was calculated as SEM × 1.96 × √ 2, where SEM is the standard error of the measurement and 1.96 is the z-score for a 95% confidence interval. Where appropriate, we measured effect sizes with Cohen’s d for repeated measures (d*_RM_*) or with matched-pairs rank-biserial correlation (r*) for the Wilcoxon signed-rank test. When Friedman ANOVA was performed, we reported Kendall’s W, as a measure of effect size. In addition to that, we also calculated the difference in motor scores between each follow-up and baseline value and expressed them as an improvement ratio (%). To estimate relationships between variables, we used linear regression analysis. To test associations between demographic variables, MRI data, and motor and non-motor scores at baseline and at follow-up visits, we employed Kendall rank correlation and partial correlation analysis. For multiple comparisons, Bonferroni correction was used. The statistical significance level was *p* < 0.05.

## 3. Results

### 3.1. Patients

Baseline demographic and clinical characteristics of patients are presented in Table 1. The majority of patients were men. Eleven (52.4%) patients had stage II of the disease according to the Hoehn and Yahr scale (range 1 to 3).

### 3.2. Follow-Up

#### 3.2.1. Tremor and Motor Assessments

##### Individual Patient Assessments

Before thalamotomy, 3 patients had moderate hand tremor and, in 18 patients, hand tremor was severe. On the 2nd day after MRgFUS, in 11 (52.4%) patients, hand tremor disappeared and, in 10 (47.6%) patients, it decreased from a median of 7.0 (IQR 5.25; 9.0) before thalamotomy to a median of 0.5 (IQR 0.0; 1.0) after the procedure. A complete reduction in leg tremor was observed in 7 (46.7%) out of 15 patients with hemitremor.

**Figure 4:**
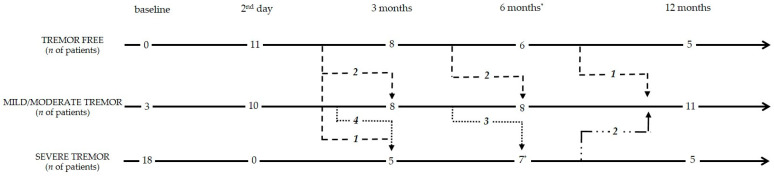
Flowchart of the evolution of tremor during follow-up. (*—medications added for tremor correction at the end of the 6th month).

The evolution of hand tremor during the follow-up is shown in Figure 4. By the end of the 12th month, five (24.9%) patients were tremor-free, which was defined as the absence of tremor. Eleven (52.4%) patients had partial improvement in hand tremor (1–4 points [item 20–21]) on the treated side. In five (24.9%) patients, there was a relapse of severe tremor (>4 points [item 20–21]). No correlation between severity of tremor at baseline and outcome at 12 months was found.

The severity of hypokinesia and rigidity decreased along with tremor reduction. On the 2nd day after surgery, the number of patients with severe bradykinesia decreased from 11 to 5 (45.5%) patients. By the end of the 12th month, the number of patients with severe bradykinesia increased to eight (72.7%). The number of patients with severe rigidity also decreased from ten patients before operation to five patients by the end of follow-up.

***Scale assessments***

**Figure 5:**
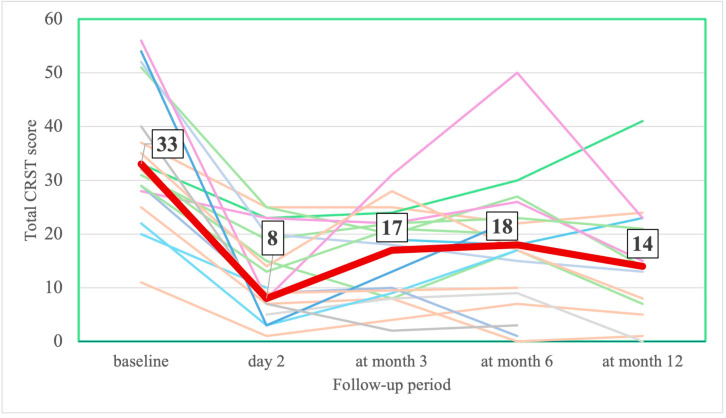
Change in individual (thin different color lines) and median (thick line) CRST score at baseline and at follow-up (number in box are median values).

**Figure 6:**
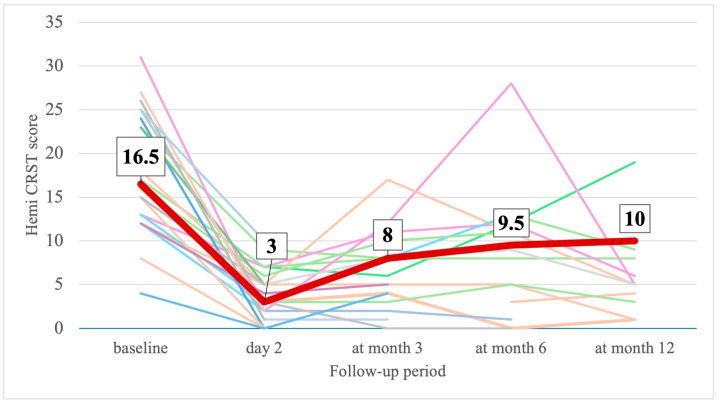
Change in individual (thin different color lines) and median (thick line) hemi-CRST score at baseline and at follow-up (number in box are median values).

**Figure 7:**
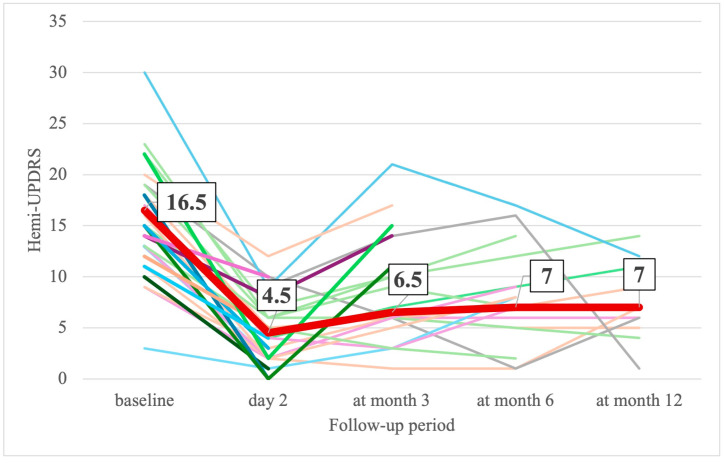
Change in individual (thin different color lines) and median (thick line) hemi-UPDRS score at baseline and at follow-up (number in box are median values).

On the 2nd day after MRgFUS, UPDRS and hemi-UPDRS scores decreased by 51% and 70% and CRST and hemi-CRST scores decreased by 62.5% and 70% (Table 2, Figure 5 and Figure 6). The total scores on part III of the UPDRS decreased by 45% (Table 2, Figure 7). By the end of the 3rd, 6th, and 12th months, UPDRS and hemi-UPDRS scores, and CRST and hemi-CRST scales, as well as rest and action tremor (UPDRS items 20–21), remained significantly decreased. The effect sizes for UPDRS, hemi-UPRDS, CRST and hemi-CRST between baseline and the end of the follow-up remained large, Cohen’s d*_RM_* ≤ −0.854.

Besides reduction in rest and action tremors, there was also a significant reduction in hypokinesia and rigidity (UPDRS item 23–26 for hypokinesia and item 22 for rigidity) on day 2 and at 3 and 6 months’ follow-up (Table 2). Effect size for hypokinesia and rigidity between baseline and the end of the 6th month remained large, Cohen’s d*_RM_* ≤ −0.804. After the 6th month, the effect of VIM ablation on hypokinesia and rigidity began to decrease and was no longer statistically significant by the end of the follow-up (Table 2).

#### 3.2.2. Non-Motor Assessment and Symptoms

***Non-Motor Symptoms Scale***. There was no statistically significant improvement in Non-Motor Symptoms Scale: Friedman ANOVA, χ^2^ = 3.4, df = 3, *p* = 0.328 (Table 3).

***QoL.*** QoL improved between baseline and the end of the 3rd month: Wilcoxon matched-pairs signed-ranks test: z = 1923, *p* = 0.0545 (effect size r* = −0.441). There was also a tendency in improvement of QoL between baseline and the end of the follow-up (Friedman ANOVA, χ^2^= 6.40, df = 3, *p* = 0.0937 (Table 3)). There was no improvement in QoL between the 3rd and 6th months or 3rd and 12th months. A linear regression analysis revealed that at baseline and by the end of the follow-up, UPDRS score, ADL, and depression determined variations in the QoL (Table 4). At all follow-up points, QoL was correlated with anxiety (Kendall τb ≥ 0.2927, *p* ≤ 0.051) and with depression (Kendall τb ≥ 0.5031, *p* ≤ 0.003). There was no correlation between QoL and tremor (Kendall τb ≤ 0.2582, *p* ≥ 0.115).

**Figure 8:**
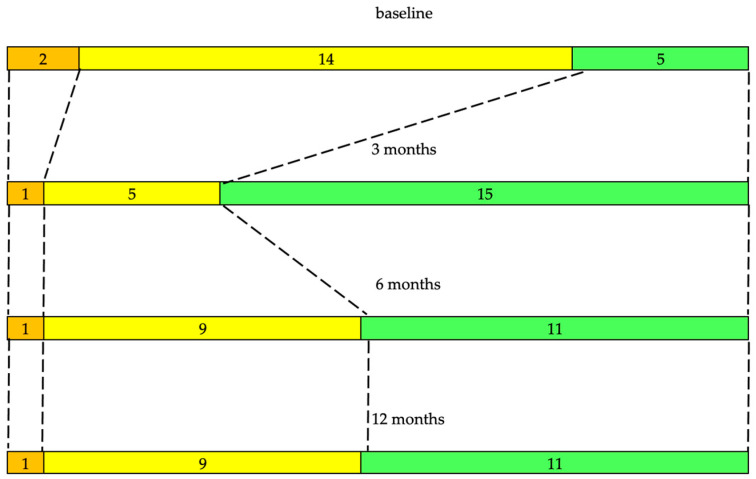
Change in activities of daily living between baseline and each follow-up (21 patients, Friedman ANOVA, χ^2^ = 13.24, df = 3, *p* = 0.0042). Orange—ADL score ≤ 50. Yellow—ADL score 60–80. Green—ADL score ≥ 90.

***ADL***. At baseline, five (23.8%) patients were completely independent based on their ADL (S&E ADL score ≥ 90). By the end of the 3rd month after thalamotomy, the number of completely independent patients reached 14 (χ^2^ = 6.15, OR = 6.40, 95% CI = 1.39–31.97, *p* = 0.0131). At follow-ups, ADL correlated with the UPDRS (Kendall τb ≥ −0.3622, *p* ≤ 0.004), CRST (Kendall τb ≥ −0.3379, *p* ≤ 0.009), and QoL (Kendall τb ≥ −0.2509, *p* ≤ 0.017). There was no correlation between ADL and tremor (Kendall τb ≤ −0.2956, *p* ≥ 0.269). The change in distribution of ADL scores between baseline and each follow-up was significant: χ^2^ = 13.24, df = 3, *p* = 0.0042 (Figure 8). The increase in ADL score between baseline and the end of the 3rd month reached 7.619 (95% CI = 1.36; 13.89, t = 2.54, df = 20, *p* = 0.02) and was of greater magnitude than the MDC of 6.04 in our sample. There was no significant improvement in the ADL after the 3rd month (Table 3).

***Anxiety.*** Anxiety progressed from the baseline to the end of the 3rd month (z = −2.13, *p* = 0.019, r* = −0.516). There was a progression of anxiety between baseline and the end of follow-up: Friedman ANOVA: χ^2^ = 12.2, df = 3, *p* = 0.007, Kendall’s W = 0.793 (Table 3). Mean paired differences between baseline and months 3, 6, and 12 were baseline–3rd month = 5.45, 95% CI = 2.19–8.70, t = 3.51, df = 19, *p* = 0.002; baseline–6th month = 4.50, 95% CI = 1.02–7.98, t = 2.71, df = 19, *p* = 0.014; and baseline–12th month = 5.81, 95% CI = 1.73–9.89, t = 2.97, df = 20, *p* = 0.008. At all follow-ups, mean paired differences were greater than the MDC of 3.8 in our study. Anxiety correlated with depression at all follow-up points (Kendall τb ≥ 0.3599, *p* ≤ 0.038).

***Depression.*** There was a tendency of depression to progress between the baseline and end of the study (Friedman ANOVA: χ^2^ = 6.5, df = 3, *p* = 0.091, Table 3). Depression at all follow-up points strongly correlated with QoL (Kendall τb ≥ 0.5031, *p* ≤ 0.003) and UPDRS score (Kendall τb ≥ 0.3526, *p* ≤ 0.041).

***Apathy***. Apathy did not significantly change throughout the study—Friedman ANOVA: χ^2^ = 3.7, df = 3, *p* = 0.301 (Table 3).

***Cognitive function.*** Total mean MoCA score at baseline was 26.0 ± 2.9 and remained unchanged throughout the study (Table 3). Still, a cohort of patients with baseline MoCA score < 26 points demonstrated an improvement during the follow-up (Table 3). Mean paired difference between baseline and the 3rd, 6th, and 12th months was 2.2 [95% CI = 0.35–4.09, t = 2.73, df = 8, *p* = 0.026], 2.9 [95% CI = 0.84–4.93, t = 3.25, df = 8, *p* = 0.012], and 3.0 [95% CI = 0.66–5.34, t = 2.96, df = 8, *p* = 0.018], respectively. Improvements were observed in attention, speech activity, delayed recall and orientation. At every follow-up, the improvement in MoCA score was of greater magnitude than the MDC (1.99) in our sample and the minimal clinically important difference (MCID) reported by Wu CY. et al. [41] and Lindvall E. et al. [42] for patients after stroke without dementia. We observed no significant difference in MoCA score between months 3, 6, and 12. In patients with baseline MoCA score ≥ 26 points, no improvement was found, which was probably due to the ceiling effect (Table 3).

***Impulsive–compulsive disorders***. Four patients had ICDs. Three patients had one ICD (punding or hypersexuality), and one patient had a combination of binge eating and DDS. Impulse control disorders (except for excessive shopping) disappeared in two patients by month 3 (one patient with binge eating and DDS, one with hypersexuality) and in one patient (punding) in 6 months but reappeared in one patient at months 12 (binge eating), though with lower severity. One patient started to overeat by the end of the 12th month.

#### 3.2.3. LEDD

Before MRgFUS, 7 (33.3%) patients received levodopa and 14 (67.3%) were taking a combined treatment (dopamine receptor agonists, amantadines, anticholinergics, clonazepam, and propranolol). During the follow-up, levodopa was added in five patients. By the end of the study, 12 (57.1%) patients were taking levodopa. Initially, 9 (42.9%) patients received anticholinergic therapy. By the end of the follow-up period, the number of patients taking anticholinergic therapy decreased to four (19.0%).

According to changes in LEDD between baseline and the end of the follow-up, we divided all patients into the following groups: “increased” LEDD group—12 (57.2%) patients; “decreased” LEDD group—4 (19.0%) patients; and “stable” LEDD group—5 (23.8%) patients. Since the number of patients in the “decreased” and “stable” LEDD groups was small, for the purpose of the statistical analysis, we combined the “decreased” and “stable” LEDD groups (Table 5).

At baseline, there was no difference in LEDD between the “increased” and “combined” groups: 461.5 ± 312.3 vs. 565.3 ± 292.9, t = 0.96, 95% CI for difference −75.5; 230.8, t = 0.77, *p* = 0.44. By the end of the follow-up, in the “increased” LEDD group, the increase in LEDD was +221 ± 87, and in the “combined” LEDD group, daily dose decreased by −63 ± 96: t = 6.20, 95% CI for difference 165.8; 407.9, *p* = 0.000.

In the “increased” LEDD group, a significant increase in LEDD was observed between baseline and months 6 and 12, as well as between months 3 and 12, and months 6 and 12 (Table 6). In contrast, in the “combined” LEDD group, LEDD decreased by the end of the 3rd month and remained stable thereafter (Table 7).

At baseline, the severity of motor and non-motor symptoms did not differ between LEDD groups (Table 8). By the end of the follow-up, in the “increased“ LEDD group, the hemi-CRST score (Mann–Whitney U-test z = −2.32; *p* = 0.020) and tremor + rigidity + hypokinesia score on the treated side (Mann–Whitney U-test z = −2.46; *p* = 0.014) were significantly higher than in the combined LEDD group. Also, there was a tendency to significant increase in UPDRS scores (Mann-Whitney U-test z = −1.82; *p* = 0.069) (Table 9). A linear regression analysis revealed that among motor and non-motor symptoms, the progression of motor symptoms (tremor + rigidity + hypokinesia) on the treated side from the 2nd day post-FUS to the end of the 6th month after FUS thalamotomy determined the increase in LEDD by the end of the follow-up period (Table 10).

Linear regression analysis (stepwise method): Variables entered in the equation at baseline, 3, 6, and 12 months: ADL, anxiety, apathy, depression, hemi-CRST treated/untreated side, progression of hemi-CRST on treated/untreated side between 2nd day after FUS thalamotomy and 12th month, LEDD, NMSS, QoL, tremor + hypokinesia + rigidity on treated/untreated side, progression of tremor + rigidity + hypokinesia between 2nd day and end of the 6th month on treated/untreated side, progression of tremor + hypokinesia + rigidity between day 2 after FUS thalamotomy and end of the follow-up on treated/untreated side, tremor severity on treated side, progression of tremor severity on treated/untreated side between 2nd day after FUS thalamotomy and 12th month, UPDRS, and progression of UPDRS score between 2nd day after FUS thalamotomy and 12th month.

#### 3.2.4. AE

We classified AE as related to thalamotomy or to FUS/MRI procedure. Thirteen (61.9%) patients had thalamotomy-related AE. The AE included hemi- or monoparesis of 4.0–4.5 points on Medical Research Council scale (n = 5), dysarthria (n = 4), hemiataxia (n = 4), orofacial (n = 4), and finger (n = 1) parasthesia or numbness (Table 11). Six (28.5%) patients had a combination of two or three AE. In four patients, AE resolved within 3 months, in three patients, within 6 months, and in two patients, within 12 months. By the end of the follow-up, four (19.0%) patients had persistent AE. Mild foot paresis, dysarthria, and oral parasthesia were observed in one patient each, and one patient had a combination of mild hemiataxia and orofacial parasthesia (Table 11). FUS/MRI-related AE were found in nine (42.9%) patients and included post-surgery dizziness (n = 3), neck pain (n = 3), and headache (n = 7). All these AE resolved by the end of the 2nd day after the procedure.

#### 3.2.5. MRI Changes

**Figure 9:**
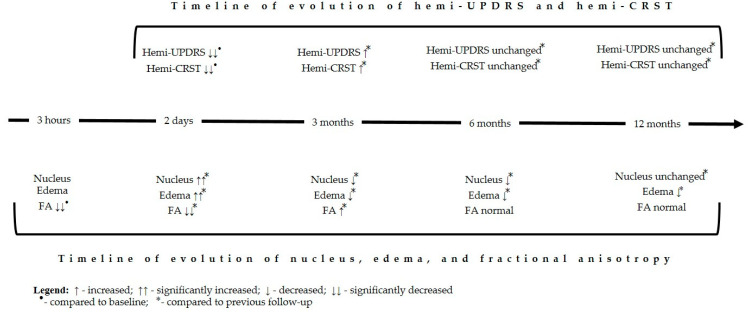
Timeline of evolution of motor symptoms and MRI change during follow-up.

**Figure 10:**
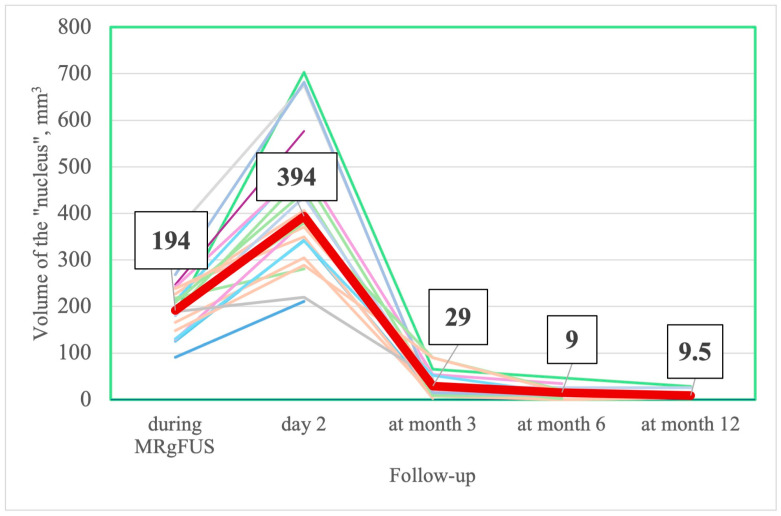
Change in individual (thin different color lines) and median (thick line) volume in “nucleus” during FUS thalamotomy and at follow-up visits (number in box are median values).

**Figure 11:**
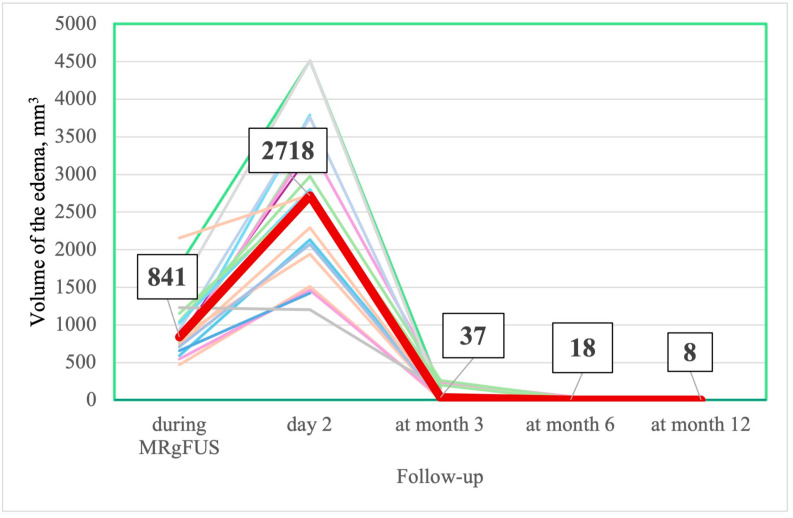
Change in individual (thin different color lines) and median (thick line) in edema volume during FUS thalamotomy and at follow-up visits (number in box are median values).

The nucleus (lesion) and edema reached their maximal values on the 2nd day after FUS thalamotomy. Thereafter, the volumes of the nucleus and edema decreased gradually, with almost complete resolution by the end of the 3rd month (Table 12, Figure 9, Figure 10 and Figure 11).

**Figure 12:**
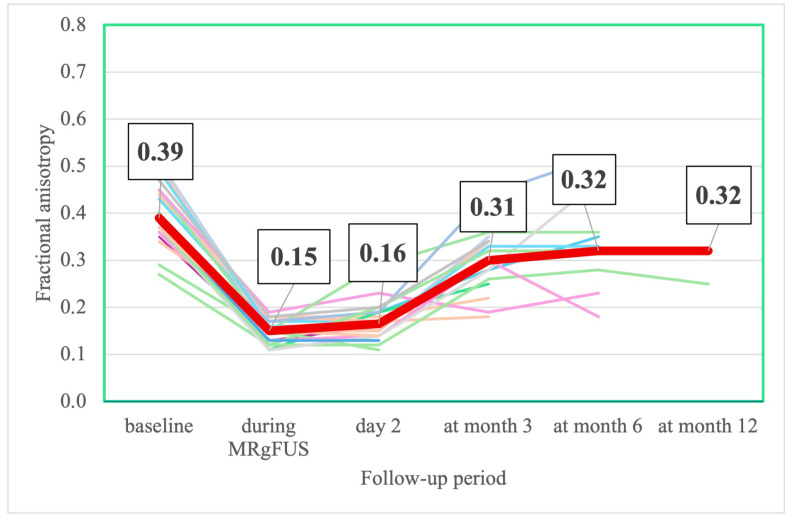
Change in individual (thin different color lines) and median (thick line) in fractional anisotropy values during FUS thalamotomy and at follow-up visits (number in box are median values).

The FA was minimal 3 h after MRgFUS. Starting from the 2nd day after FUS thalamotomy, FA values began to increase gradually. By the end of the 6th month, there was no significant difference from the preoperative values (Table 11, Figure 12).

There were no statistically significant associations between motor or non-motor symptoms and MRI changes.

## 4. Discussion

Treatment of pharmacoresistant tremor is a challenging task. At present, DBS and ablation of VIM by FUS are used for correction of tremor. MRgFUS thalamotomy is a relatively new technology of incisionless targeted ablation of the VIM thalamus. Recent research has shown efficacy of unilateral FUS thalamotomy in reducing pharmacoresistant tremor in TDPD patients. Data from systematic reviews and meta-analyses show comparable efficacy between MRgFUS VIM thalamotomy and VIM DBS [43,44]. Cesarano S. et al. demonstrated that staged bilateral FUS thalamotomy is also effective in alleviating bilateral tremor [45]. Besides the VIM thalamus, other targets for treating drug-induced dyskinesias and other motor symptoms in PD patients are now being studied [14,16,46].

This study has shown the lasting effect of MRgFUS VIM thalamotomy on reduction in tremor in TDPD patients. The effect on tremor reduction was most significant on the 2nd day after the procedure. By the end of the 12th month, five patients remained tremor-free. In 11 patients, mild/moderate hand tremor re-emerged; still, the symptoms remained below their pre-operational level. In total, by the end of the follow-up, 16 (76.2%) patients demonstrated significant improvement in hand tremor. Five (23.8%) patients had a relapse of severe hand tremor. Previous studies have also shown the re-emergence of hand tremor and the diminishing effect of MRgFUS on motor symptoms after 6–12 months [15]. Size of lesion and thalamic targeting may appear as the relevant factors for tremor relapse after FUS thalamotomy in TDPD patients [47]. Braccia A. et al. [48] demonstrated that relapse of tremor at 1 month and 6 months’ follow-up was associated with smaller volume lesion within 24 h after the procedure, shorter disease duration, younger age, and lower total MDS-UPDRS-III scores in an “on medication” state. Also, reorganization of functional networks for tremor after thalamotomy, as was shown in patients with ET, may result in re-emergence of tremor [49]. Besides that, mechanisms of tremor in PD patients are not solely determined by nigrostriatal dopaminergic deficiency but involve dysfunction of serotonergic, dopaminergic, and noradrenergic systems and anatomical structures such as the cerebellum, dentatorubrothalamic tract, thalamic nuclei, primary motor, and premotor cortices [50,51,52].

We also found that VIM thalamotomy was effective in alleviation of bradykinesia and rigidity. Bradykinesia and rigidity improved immediately after the thalamotomy and remained unchanged during the follow-up. To the best of our knowledge, only a few studies have assessed the evolution of bradykinesia and rigidity after FUS thalamotomy [53,54,55]. In one study [55], statistically significant improvement in bradykinesia and rigidity on the treated side was found 1–3 days and 6 and 12 months after FUS. In two other studies, rigidity decreased significantly on 1 day, 3 months [54], and 6 months [53] after FUS thalamotomy. Several factors can potentially contribute to the transient alleviation of bradykinesia and rigidity after FUS thalamotomy. Despite the VIM thalamus being a usual target for tremor suppression in ET or PD, locating it by conventional MRI or atlas-derived techniques may be challenging [56,57]. Anatomically, the ventral lateral nucleus (VL) of the thalamus is segregated into several functionally distinct areas, including the ventral intermediate (VIM), ventral oral posterior (VOP), and ventral oral anterior (VOA) nuclei [58]. Recent studies suggest that VOP and VOA are not separate nuclei but constitute a single entity [59,60]. The VIM nucleus receives motor fibers from the cerebellum and then projects them to the primary motor cortex, while the VOP/VOA nuclei act as a relay station between the globus pallidus internus (GPi), primary and supplementary motor cortices, and dorsolateral prefrontal cortex. Both the VIM and VOA/VOP nuclei are intimately interrelated and are believed to have overlapping projections [61,62]. Thus, targeting more oral structures of the VIM nucleus by FUS may affect the projections from GPi to the VOA/VOP nuclei and, because of edema and white matter disruption, transiently alleviate bradykinesia and rigidity [63].

Recent studies demonstrated the efficacy of MRgFUS targeting of the subthalamic nucleus (STN) and GPi in reducing bradykinesia, rigidity, and the UPDRS Part III total score [44,64,65]. However, due to the limited number of studies, ablation of STN has not yet been approved by the FDA. GPi ablation by MRgFUS was approved in 2021, although the number of publications remains limited.

Besides motor assessment, we prospectively investigated the evolution of non-motor symptoms, including QoL, ADL, and cognitive and emotional/affective disorders.

***QoL.*** Few studies have investigated the effect of VIM ablation by FUS on quality of life. We observed a positive effect of FUS thalamotomy on QoL. QoL significantly improved during the first 3 months. The improvement in QoL in our study exceeded the MCID of 4.72 points reported by Horváth K. et al. for patients with PD [66]. There was no significant change in Qol after the 3rd month of follow-up. A recent systematic review and meta-analysis encompassing 66 patients with TDPD showed improvement in their QoL at the 6-month follow-up after FUS thalamotomy (standardized mean difference −0.86, 95% CI: −1.21; −0.50, *p* < 0.01) [67]. Sinai A. et al. [15] found that the positive effect of FUS thalamotomy on QoL was very marked several months after the procedure and began to decrease thereafter.

Interestingly, at baseline and at follow-up, we did not observe a significant association between QoL and tremor. This is consistent with previous research [18], which demonstrated that anxiety, mood, and behavior disorders affect QoL more than postoperative improvements in tremor severity.

In our study, progression of anxiety, depression, and motor symptoms on the untreated side and relapse of tremor on the treated side explained the absence of improvement in QoL after the 3rd month. Sperling SA. et al. [20] reported similar results. They found that 3 and 12 months after VIM ablation, worse QoL was associated with motor symptom severity on UPDRS-III and progression of non-motor symptoms—anxiety, depression, apathy, disinhibition, and executive and cognitive dysfunction. In a recent study, Purrer V. et al. [55] observed that 12 months after MRgFUS, both QoL and ADL correlated positively with the disability scales MDS-UPDRS I and II and score on the Non-Motor Symptoms Questionnaire, as well as depressive symptoms on Beck’s Depression Inventory and anxiety on the State-Trait Anxiety Scale. In a prospective study, Shi Y. et al. [68] demonstrated that in patients with PD, anxiety and depression contributed significantly to poor QoL, ADL, and reduced interval to the initiation of dopamine replacement therapy. Taken together, relapse or worsening of motor and non-motor symptoms several months after FUS thalamotomy may offset any further improvements in quality of life.

***ADL***. For assessment of ADL, we used the Schwab and England ADL Scale. The S&E ADL scale is a standard assessment tool for PD patients [33] and provides reliable information of patients’ level of independence in performing everyday activities [69]. In our study, we found significant improvements in ADL 3 months after thalamotomy. Our results are consistent with previous research [20], which shows significant improvements in self-rated ADL after FUS thalamotomy. The increase in ADL score was of greater magnitude than MDC in our study. By the end of the 3rd month, the number of completely independent patients was significantly higher than before MRgFUS. There was also a significant shift in the distribution of ADL scores between baseline and 3 month follow-up, with most of the patients moving from the partially dependent (60–80 points) category to the independent (90–100 points) category. After the 3rd month, we observed no improvement in the ADL score. A recent study [55] also demonstrated that, after ablation of VIM, improvement in QoL measured by PDQ-39 and activities of daily living assessed by the Functional Activities Questionnaire occurred within 3 to 6 months and remained stable until the end of the follow-up period.

Throughout this study, ADL significantly correlated with scores on the UPDRS, CRST, and QoL, but not with tremor. This finding may suggest that general motor impairments (postural instability, rigidity, bradykinesia, etc.) affect ADL more so than tremor itself. Earlier, Hariz GM. and Forsgren L. [70] observed that patients with the postural instability-gait difficulties subtype of PD had already had, at their first visit, a worse ADL and QoL than patients with the tremor-dominant type. To conclude, our research demonstrated that, after FUS thalamotomy, improvement in a wide range of motor and non-motor symptoms rather than just in tremor led to better activities of daily living and quality of life.

***Anxiety and depression***. Few studies have assessed the evolution of anxiety after VIM thalamotomy [55,71]. In our study, anxiety progressed from the baseline to the end of the follow-up period. At every follow-up, the mean change from baseline exceeded the minimal detectable change. Besides progression of anxiety, we also observed a tendency to progression of depression. Both symptoms correlated with each other at all follow-up points (Kendall τb ≥ 0.3599, *p* ≤ 0.038). As in previous studies [68], anxiety and depression significantly correlated with QoL (Kendall τb ≥ 0.5031, *p* ≤ 0.003) and UPDRS score (Kendall τb ≥ 0.3526, *p* ≤ 0.041).

The progression of anxiety in our study can be linked to a combination of factors. First of all, dissociation between expectations before FUS thalamotomy and relapse of tremor on the treated side or progression of symptoms on the untreated side may underlie the progression of anxiety. Besides that, the spreading of dopaminergic dysfunction because of the advancement of the neurodegeneration may contribute to the progression of anxiety. Previously, it has been shown that advancement of dopaminergic dysfunction to the mesolimbic system and increasing imbalance between functional circuits for fear and anxiety may stimulate the progression of anxiety in patients with PD [72]. In Parkinson’s disease, dopaminergic systems show gender-specific vulnerability. Boccalini C. et al. [73] studied 286 (97 female) newly diagnosed and drug-naïve idiopathic PD patients and showed that females had significantly higher anxiety score on the State-Trait Anxiety Inventory, lower ^[123I]^FP-CIT bindings in several limbic areas, and more massive reconfiguration of the mesolimbic system’s connectivity. Lower SUVr in the amygdala was associated with high anxiety levels in females but not in males. In a recent study, Carey G. et al. demonstrated that anxiety in PD is linked to microstructural alterations within the white matter of the tracts involved in anxiety-related neuronal circuits [74]. De Micco R. et al. [72] showed that drug-naïve, cognitively unimpaired PD patients with clinically significant anxiety symptoms demonstrated decreased connectivity within the default mode and sensorimotor networks, increased connectivity within the executive control network, and both increased and decreased connectivity within the salience network and the fronto-parietal network. Dan R. et al. [75] identified three distinct types of functional connectivity patterns in PD patients with anxiety. The first type included functional connections between the orbitofrontal cortex (OFC) and temporal–limbic regions and was positively associated with anxiety. The second type was negatively associated with anxiety and included functional connections between the dorsolateral prefrontal cortex and paralimbic–limbic–OFC regions. The third type included functional connections of the sensorimotor cortex mainly with the OFC and was negatively associated with anxiety. Since the third type of associations has not been reported in idiopathic anxiety, the authors suggested that it may be a unique characterization of anxiety in PD and inherently related to motor dysfunction. Previous studies showed that, in PD patients, anxiety and depression are comorbid and anxiety symptoms at an earlier point can predict the development of depressive symptoms later on [76]. Lin H. et al. [77] determined that, in patients with advanced PD, changes in resting state functional connectivity in the insula, hippocampus, amygdala, posterior cingulate cortex, superior parietal lobule, and medial prefrontal cortex significantly correlated with the presence and severity of depression. Of note, brain regions such as the amygdala, insula, medial prefrontal cortex, and posterior cingulate cortex are also significantly associated with anxiety. In patients with Parkinson’s disease, progression of dopaminergic degeneration may contribute to the overlap between functional networks for anxiety and depression [75].

***Cognitive assessment***. At present, short- and long-term effects of FUS thalamotomy on cognitive function remain largely underexplored. Compared to other techniques, FUS thalamotomy is associated with a safer cognitive outcome, probably due to intraoperational monitoring, precise navigation, and smaller lesions. In a meta-analysis encompassing 4 studies and 72 patients (52 with ET, 20 with PD), Rohringer CR. et al. [78] concluded that unilateral MRgFUS VIM ablation was relatively safe from a cognitive standpoint. Petersen J. et al. [79], after unilateral VIM ablation, longitudinally assessed various cognitive domains in two cohorts of patients with ET (55 patients). After 4.5–6.0 months, individual patient analysis demonstrated that 24 patients showed improvement on at least one cognitive measure while 24 patients showed decline on at least one cognitive measure. Sperling SA. et al. [20] demonstrated that, in TDPD patients, unilateral VIM ablation with FUS did not worsen cognitive functions and did not affect mood over a period of 3 or 12 months post-surgery.

In our study, we did not observe a negative impact of FUS thalamotomy on cognitive functions. Moreover, in a cohort of patients with baseline MoCA < 26 (mean 22.9 ± 1.4) there was a significant improvement in MoCA score between baseline and the end of the 3rd month. The mean difference in MoCA between baseline and the end of the 3rd month reached 2.2 and was greater than the MDC in our sample. In this cohort, there were no significant changes in MoCA score after the 3rd month.

Other studies have also demonstrated short-term benefits of FUS thalamotomy on cognitive functions in patients with PD or ET. Jung NY. et al. [11] showed that, 6 months after left-sided FUS thalamotomy, patients with ET had an improved performance on the Seoul Neuropsychological Screening Battery II (Korean version of the Boston Naming Test and memory functions). In another study, in PD patients, a significant improvement was demonstrated in MoCA scores (22.56 ± 4.10 and 23.94 ± 3.65, *p* = 0.003) and a tendency for improvement in MMSE scores (25.70 ± 3.78 and 27.31 ± 2.44, *p* = 0.072) by the end of the 6th month after left-sided FUS thalamotomy [22]. Similar improvements in MoCA scores (25.7 ± 3.1 at baseline vs. 26.3 ± 2.6 at the end of the 12th month, Z = −2.25, *p* = 0.024) were found by Purrer V. et al. [55]. In contrast, Bond AE. et al. [19] and Sperling A. et al. [20], who studied patients with TDPD and baseline median MoCa score of 25.5 (IQR = 23–27.5), did not observe changes in MoCA scores 3 or 12 months after FUS ablation of the VIM thalamus (Me 25.5, IQR = 23.3–26.8, and Me 25.0, IQR = 24.0–26.0, respectively). We also, in a cohort of patients with baseline MoCA score ≥ 26 (mean MoCA = 28.1 ± 1.5), did not find improvements in MoCA scores. In our study, the lack of improvement was probably due to a ceiling effect.

Several factors may contribute to cognitive outcomes after unilateral FUS thalamotomy. Saporito G. et al. [71] observed distinct patterns of cognitive improvement after VIM ablation depending on the left/right hemisphere. Patients with a left VIM FUS ablation demonstrated significant improvement in memory and frontal functions. In patients with right VIM thalamotomy, an improvement in anxiety, depression, and QoL was observed [71].

The improvement of cognitive functions after FUS thalamotomy also can be linked to reorganization of the cerebellar-subcortical-cortical networks. Despite the VIM nucleus being traditionally viewed as a motor nucleus, recent research suggests that it may contribute to cognitive functions though its connections to the cerebral cortex via thalamocortical pathways [80]. Ablation of the VIM nucleus by FUS can reorganize brain functional connectivity, including motor-related, visuomotor, and default-mode networks. Wen L. et al. [81] demonstrated that unilateral ablation of the VIM nucleus caused alterations in regional homogeneity in various brain regions, including the frontal and temporal lobes and hippocampus, areas which are critical for cognitive functions. In a study by Dahmani L. et al. [82], the brain regions most affected by connectivity changes after unilateral FUS thalamotomy mainly belonged to the default mode and limbic networks as well as to dorsal and ventral attention networks, which play a key role in attention, orientation, memory, and motivation.

Another possible explanation for cognitive improvement after FUS thalamotomy may be a transient opening of the blood–brain barrier (BBB). Cognitive decline in patients with Parkinson’s disease is associated with a combination of pathologies, including alpha-synuclein aggregates and beta-amyloid plaques. While abnormal accumulation of alpha-synuclein aggregates is the primary driver of cognitive symptoms in patients with PD, amyloid deposition can accelerate cognitive deficit [83]. Previous studies in patients with Alzheimer’s disease suggest that region-specific transient opening of BBB by FUS may potentially relieve local amyloid deposition and improve cognitive functions several months after the procedure [84,85]. In patients with PD and dementia (baseline MMSE score 18.11 ± 3.9) and increased Aβ-binding on ^[18F]^-Flutemetamol PET scan, Gasca-Salas C. et al. [86] demonstrated that repeated opening of the BBB in the parieto-occipito-temporal area led to improvement on the MMSE test (21.6 ± 3.4), the Stroop test, short-term verbal and visual memory, recognition, and visuospatial function 3–4 weeks after the second opening. Despite improvements in cognitive tests, there were no significant changes in amyloid or glucose uptake patterns pre- to post-treatment. In another study [87], in seven PD patients with cognitive impairment, repeated opening of the BBB in the posterior putamen significantly reduced amyloid uptake in the targeted region but did not change ^18^F-FDOPA PET nor improve cognitive functions. BBB opening by FUS may also initiate change in regional cerebral glucose metabolism and thus have beneficial effects on cognitive functions. Jeong H. et al. [88] demonstrated that low-intensity FUS with the right hippocampus as the target area in patients with AD dementia caused mild improvements in memory, executive, and global cognitive functions and significantly (*p* < 0.001) increased glucose metabolism in the temporal, cingulate, and frontal cortices after two weeks’ follow-up. Further studies are necessary to understand how the location, timing, and duration of BBB opening influence cognitive functions.

Physical inactivity and a sedentary lifestyle may also contribute to cognitive impairment in patients with PD. A recent systematic review (7 observational studies, 980 participants) demonstrated that, in patients with PD, higher amounts of both objectively measured and self-reported sedentary time were associated with greater impairments in memory, concentration, verbal fluency, and executive function [89]. In contrast, regular physical exercise was associated with improvements in global cognitive function (SMD = 0.69; 95% CI = 0.31 to 1.06; *p* < 0.001) [90]. In a longitudinal observation study, Diaz-Galvan P. et al. [91] showed that, in patients with early PD, slower decline in memory and attention was mediated by slower decreases in temporal and parietal cortical thickness and hippocampal volume.

After FUS thalamotomy, most patients show significant improvement in motor function and can return to daily activities and lead a more active lifestyle. A more active lifestyle and physical activity stimulate neuroplasticity and expression of brain-derived neurotrophic factor and other neurotrophins, modulate neuroinflammation, and improve cerebral blood flow (CBF), which, in turn, may result in improvement in cognitive functions [92,93,94]. Lin L. et al. [95] demonstrated that, in older people with mild cognitive impairment (MCI), even 30 min of low-intensity walking administered for 12 weeks induced significant increases in serum BDNF and improvement in MoCA. Regular physical activity may offer more cognitive benefits to people with MCI than to cognitively normal individuals or to patients with dementia. In a 6-year follow-up of 247,149 individuals, Kim YJ. et al. [96] demonstrated that regular aerobic moderate-intensity physical activity (>5 days/week) or vigorous-intensity physical activity (>3 days/week) prevented conversion from MCI to dementia of Alzheimer’s type. Demurtas J. et al. [97], in a meta-analysis (28,205 participants, 14,209 physical activity group), showed that mixed physical activity or resistance training interventions improved global cognition both in people with MCI and in people with dementia, while improvements in executive function, attention, and memory were found only in individuals with MCI.

***LEDD***. In our study, after FUS thalamotomy, patients continued taking medications for suppression of rigidity and bradykinesia. According to changes in LEDD between baseline and the end of follow-up, all patients were divided into “increased” and “combined (decreased + stable)” LEDD groups. By the end of the follow-up, in 9 (42.9%) patients, levodopa equivalent daily dose remained stable or decreased compared to baseline, while in 12 (57.1%) patients, LEDD was increased.

Levodopa equivalent daily dose decreased between baseline and the end of the 3rd month; after that, the LEDD remained stable. In contrast, in the “increased” LEDD group, the mean LEDD did not significantly change between baseline and the end of the 3rd month but increased between baseline and the end of the 6th and 12th months. A significant determinant of the increase in LEDD by the end of the follow-up was the progression of motor symptoms (tremor + rigidity + hypokinesia) on the treated side in the 6th month after FUS thalamotomy. Earlier, several studies reported stabilization or decrease in LEDD after FUS thalamotomy. Bond AE. et al. [19] observed decreases in median LEDD 1, 3, and 12 months after FUS thalamotomy. Purrer V. et al. [55] demonstrated a decrease in LEDD in 32% of patients by the end of the 12th month. In contrast, a study of 11 patients with TDPD who underwent FUS thalamotomy demonstrated an increase in LEDD in 30% of patients by the end of the 12th month [21]. Sinai A. et al. [15] and Andreasi N. et al. [53] observed stabilization of LEDD after FUS thalamotomy.

***MRI change***. The trend in the MRI change in the volume of the nucleus and edema was similar in all patients. In our sample, the maximal values of the nucleus and edema were significantly larger than in the study of Zur et al. [98]. Different methods of nucleus and edema calculation may explain this finding. We manually delineated the nucleus and edema, while Zur et al. automatically subtracted previously normalized images from an average brain model (Montreal Neurological Institute). In a study by Boecker H. et al., delineation was performed manually, and the size of the “nucleus” was very close to ours, while the edema was assessed on T2-weighted images and was smaller [99]. The FA values in the ablation region (“nucleus”) decreased significantly immediately after FUS thalamotomy, likely due to the disruption of neural tracts [100]. FA values in the “nucleus” at months 6 and 12 and in the edema area at months 3, 6, and 12 demonstrated a highly variable range, presumably due to the small size of the lesions and their proximity to the resolution limit of FA maps, thus rendering their values unreliable [101].

***AE***. Thirteen (61.9%) patients had thalamotomy-related AE. Transient mild mono- or hemiparesis, dysarthria, orofacial parasthesia, and ataxia were the most common symptoms and most of them resolved by the end of the follow-up. Persistent AE were observed in four (19.0%) patients. Previous studies have also reported a similar spectrum, frequency, and evolution of thalamotomy-related AE [15,19,53]. Besides thalamotomy-related AE, nine (42.9%) patients reported FUS/MRI-related adverse events. They included post-surgery dizziness, neck pain, and headache and resolved by the end of the 2nd day after the procedure.

***Limitations***. Our study has several limitations. It was an open-label single-center study without a control group. Small sample size limits the ability to generalize our findings to a broader population of patients with TDPD. We also acknowledge the 12-month follow-up restriction, the lack of a standardized protocol for medication adjustments, and the use of manual delineation for quantifying MRI lesions and edema. We also note the limited assessment of non-motor symptoms, specifically the lack of in-depth analysis of anxiety and depression progression and the absence of structured psychiatric evaluations.

***Future directions.*** Future research should prioritize large-scale, multi-center, randomized controlled trials with long-term follow-up. Studies assessing feasibility, safety profile, and clinical benefit of staged bilateral lesions would address significant unmet needs for patients with symmetric or bilateral symptoms. Furthermore, the scope of research must expand beyond tremor control. While the thalamus (VIM) is the established target for MRgFUS, other potential targets—the GPi, STN, and pallidothalamic tract—should be explored, and short- and long-term outcomes should be analyzed. There is also a pressing need for studies designed to evaluate the impact of MRgFUS on other motor symptoms (such as bradykinesia and rigidity), as well as non-motor symptoms. A comprehensive understanding of FUS’s effect on this wide spectrum of parkinsonian symptoms is crucial for defining its full therapeutic potential.

## 5. Conclusions

The results of our 12-month prospective study of 21 patients with TDPD demonstrated the efficacy of FUS thalamotomy in reducing tremor, hypokinesia, and rigidity, as well as in improving QoL, ADL, and cognitive functions. Most of the improvements were observed within the first 3 months after FUS thalamotomy and afterwards remained stable or started to diminish. AE were mostly transient, thus confirming the safety of FUS thalamotomy in TDPD patients. By the end of the follow-up period, FUS thalamotomy did not prevent progression of motor and non-motor symptoms or increase in levodopa equivalent dose in most of the patients.

The advantages of MRgFUS, compared to DBS, include its minimal invasiveness, immediate onset of effect, absence of age restrictions (including those associated with cognitive impairment), reduced surgical risks, favorable safety profile, and lack of implanted hardware or the need for repeated hospital visits for stimulator adjustments, as well as greater cost-effectiveness [102,103,104]. As MRgFUS is currently only approved unilaterally for PD, a patient with significant bilateral symptoms may be a better candidate for DBS. Potential limitations of MRgFUS comprise possible tremor recurrence, the irreversibility of the brain lesion, and the inability to adapt the therapy to disease progression.

## Figures and Tables

**Table 1:** Baseline demographic and clinical characteristics of study patients (*n* = 21).

Study Parameter	Value
Age, median [IQR ^a^], y	61 [54; 67]
Men/women, No. (%)	14 (66)/7 (34)
Disease duration, median [IQR], y	7 [4.5; 8.5]
Duration of antiparkinsonian medications, median [IQR], y	6 [4.5; 8.5]
Operated hemisphere: right/left, No. (%)	12 (57)/9 (43)
H & Y ^b^ stage (1/1.5/2/2.5/3), No.	1/2/11/5/2
NMSS ^c^ (<10 points/10–20 points/21–30 points/>30 points); No. (%)	11 (52.4%)/4 (19%)/5 (23.8%)/1 (4.8%)
Cognitive function, MoCA ^d^ (normal/MCI ^e^), No. (%)	11 (52.4)/10 (47.6)
Impulsive–compulsive disorder (QUIP ^f^) (absent/present), No. (%)	4 (19)/17 (81)
Apathy (absent/present), No. (%)	4 (19)/17 (81)
Depression (absent/mild), No. (%)	10 (47.6)/11 (52.4)
Anxiety (low/mild), No. (%)	20 (95.2)/1 (4.8)
Quality of life (score >50/score 25–50/score < 25), No. (%)	7 (33.3)/6 (28.6)/8 (38.1)
Activity of daily living mostly dependent (≤50)/partially dependent (60–80)/independent (90–100), No. (%)	2 (9.6)/14 (66.6)/5 (23.8)

^a^ IQR—interquartile range, ^b^ H & Y—Hoehn and Yahr scale, ^c^ NMSS—non-motor scale score, ^d^ MoCA—Montreal Cognitive Assessment, ^e^ MCI—mild cognitive impairment, ^f^ QUIP—Questionnaire for Impulsive–Compulsive Disorders in Parkinson’s Disease.

**Table 2:** Change in motor symptoms in PD patients at baseline and during follow-up.

Variables	Before MRgFUS	Day 2 Post-MRgFUS	3 Months Post-MRgFUS	6 Months Post-MRgFUS	12 Months Post-MRgFUS
UPDRS ^a^, median [IQR ^b^]	37.0 [24.5; 47.0]	18.0 [12.0; 31.0] ^#^	25.0 [17.5; 37.0] ^#^	26.0 [17.5; 32.5] *	26.0 [19.0; 32.0] ^†^
UPDRS, % reduction from baseline		51% [95% CI 34–67%] ^#^	33% [95% CI 14–49%] ^#^	30% [95% CI 16–46%] ^#^	30% [95% CI 15–48%] ^#^
UPDRS III, median [IQR]	23.5 [14.0; 35.75]	13.0 [7.0; 18.0] ^#^	14.5 [11.25; 21.5] ^#^	14.0 [10.5; 22.5] ^#^	17.0 [13.0; 25.0] ^#^
UPDRS III, % reduction from baseline		45% [95% CI 28–63%] ^#^	37% [95% CI 15–46%] ^#^	32% [95% CI 7–56%] ^#^	33% [95% CI 11–55%] ^#^
Hemi-UPDRS ^c^, median [IQR]	16.5 [12.0–19.75]	4.5 [2.0–8.25] ^#^	6.5 [6.0–13.0] ^#^	7.0 [5.0–10.5] ^#^	7.0 [5.5–11.5] ^#^
Hemi-UPDRS, % reduction from baseline		70% [95% CI 60–78%] ^#^	57% [95% CI 37–74%] ^#^	60% [95% CI 39–84%] ^#^	52% [95% CI 29–74%] ^#^
CRST ^d^, median [IQR]	33.0 [25.0–47.0]	8.0 [3.0–20.0] ^#^	17.0 [6.0–23.0] ^#^	18.0 [12.0–28.5] ^#^	14.0 [6.0–22.5] ^#^
CRST, % reduction from baseline		62.5% [95% CI 48–76%] ^#^	48% [95% CI 34–61%] ^#^	39% [95% CI 17–55%] ^#^	44% [95% CI 31–65%] ^#^
Hemi-CRST ^e^, median [IQR]	16.5 [13.0–25.0]	3.0 [2.0–5.0] ^#^	8.0 [4.0–12.0] ^#^	9.5 [3.5–12.75] ^#^	10.0 [4.0–13.0] ^#^
Hemi-CRST, % reduction from baseline		70% [95% CI 54–85%] ^#^	50% [95% CI 23–76%] ^#^	44% [95% CI 13–75%] ^#^	41% [95% CI 17–71%] ^#^
Rest tremor + action tremor on UPDRS in the operated side (item 20–21), median [IQR]	7.0 [5.0–9.0]	1.0 [0.0–1.0] ^#^	3.0 [1.0–6.0] ^#^	4.0 [0.0–5.0] ^#^	3.0 [1.0–4.0] ^#^
Rest tremor + action tremor in the operated side (item 20–21), % reduction from baseline		85% [95% CI 63–96%] ^#^	62% [95% CI 39–88%] ^#^	53% [95% CI 26–81%] ^#^	59% [95% CI 32–77%] ^#^
Rigidity on UPDRS (item 22), median [IQR]	3.0 [2.0–4.0]	1.0 [0.0–2.0] ^#^	1.0 [0.0–3.5] ^#^	1.0 [0.0–2.0] ^#^	2.0 [1.0–3.0] ^#^
Rigidity on UPDRS (item 22), % reduction from baseline		62% [95% CI 28–96%] ^#^	63% [95% CI 16–98%] ^#^	66% [95% CI 22–98%] ^#^	34% [95% CI 16–57%] ^#^
Hypokinesia on UPDRS (items 23–26), median [IQR]	5.5 [2.25–9.0]	4.0 [1.0–4.5] ^#^	4.0 [2.0–6.75] ^#^	3.0 [1.0–4.0] ^#^	4.0 [3.0–5.0]
Hypokinesia on UPDRS (items 23–26), % reduction from baseline		24% [95% CI 11–38%]	23% [95% CI 8–40%] ^#^	36% [95% CI 17–54%]	24% [95% CI 10–36%]

^a^ UPDRS—Unified Parkinson’s Disease Rating Scale, ^b^ IQR—interquartile range, ^c^ Hemi-UPDRS—UPDRS part III, items 20–26, ^d^ CRST—Clinical Rating Scale for Tremor, ^e^ Hemi-CRST-Clinical Rating Scale for Tremor items 5–6, 8–9, 11–14, ^#^—*p* ≤ 0.023, *—*p* = 0.063, ^†^—*p* = 0.078.

**Table 3:** Non-motor symptoms in PD patients at baseline and during follow-up.

Scale	Before MRgFUS	3 Months Post-MRgFUS	6 Months Post-MRgFUS	12 Months Post-MRgFUS
NMSS ^a^	7 [4.5; 23]	9 [5.5; 18.5]	11 [4.5; 20.0]	11 [8.5; 22.0]
PDQ-39 ^b^	43.0 [21.0; 57.0]	33.0 [16.5; 51.5]	34.0 [16.0; 53.5]	32.0 [13.0; 52.5]
Schwab and England Activities of Daily Living Scale	80 [75; 85]	90 [80; 90]	90 [70; 90]	90 [75; 90]
Beck Anxiety Inventory	6.0 [4.0; 10.5]	12.5 [7.25; 19.0] *	10.5 [6.25; 20.0] *	10.0 [6.5; 19.5] *
Beck Depression Inventory	10.0 [5.0; 15.5]	11.0 [7.0; 15.0]	14.0 [7.0; 16.0]	11.0 [8.0; 15.0]
Apathy Scale	9.0 [7.5; 12.0]	10.0 [7.0; 13.5]	10.0 [8.0; 13.0]	9.0 [6.5; 15.0]
MoCA ^c^ total	26.0 ± 2.9	26.6 ± 2.8	27.0 ± 2.0	26.7 ± 2.7
MoCA < 26 (n = 10)	23.1 ± 1.2	25.3 ± 2.2 ˟	26.0 ± 2.1 ˟	26.1 ± 2.7 ˟
MoCA ≥ 26 (n = 11)	28.1 ± 1.5	27.6 ± 2.9	27.8 ± 1.5	27.2 ± 2.7

˟—significant differences between baseline and months 3, 6, and 12: t ≥ 2.82, *p* ≤ 0.037, *—significant differences between baseline and months 3, 6, and 12: z ≤ −2.13, *p* ≤ 0.033. ^a^ NMSS—Non-Motor Scale Score, ^b^ PDQ-39—Parkinson’s Disease Quality of Life Questionnaire-39, ^c^ MoCA—Montreal Cognitive Assessment.

**Table 4:** Motor and non-motor symptoms determining variations in QoL (only significant results are shown).

Independent Variables	B	95% CI	T	*p*
Baseline				
Depression	1.5589	0.2784; 2.8395	2.569	0.0199
UPDRS	0.6696	0.2398; 1.0994	3.287	0.0043
12 months				
Depression	3.4349	1.8033; 5.0666	4.463	0.0004
ADL	0.7900	0.0329; 1.5471	2.212	0.0419

Linear regression analysis (stepwise method). Independent variables entered in the equation at baseline and 12 months: ADL, anxiety, apathy, depression, hemi-CRST treated/untreated side, LEDD, NMSS, tremor + hypokinesia + rigidity treated/untreated side, tremor severity treated/untreated side, UPDRS.

**Table 5:** Mean LEDD in “increased” and “combined” LEDD groups at baseline and during follow-up.

Variable	Baseline	3 Months	6 Months	12 Months
“increased” LEDD group (n = 12)				
LEDD	461.5 ± 312.3	512.7 ± 295.0	565.6 ± 252.6 *	682.3 ± 279.3 *
“combined” LEDD group (n = 9)				
LEDD	565.3 ± 292.9	500.8 ± 257.4	508.3 ± 266.3	502.7 ± 275.1

*—significant difference between baseline and months 6 and 12, t ≥ 2.56, *p* ≤ 0.026.

**Table 6:** Paired mean difference and 95% confidence intervals of LEDD between baseline and follow-up in the “increased” group (df = 11).

Time Interval	Paired Difference	95% CI	T	*p*
baseline vs. month 3	51.2	−24.2; 126.3	1.49	0.163
baseline vs. month 6	104.2	14.8; 193.6	2.56	0.026
baseline vs. month 12	220.8	165.3; 276.4	8.76	0.000
month 3 vs. month 6	52.9	−32.8; 138.6	1.36	0.201
month 3 vs. month 12	169.6	103.4; 235.9	5.64	0.000
month 6 vs. month 12	116.7	56.8; 176.5	4.29	0.001

**Table 7:** Paired mean difference and 95% confidence intervals of LEDD between baseline and follow-up in the “combined” (decreased + stable) group (df = 8).

Time Interval	Paired Difference	95% CI	T	*p*
baseline vs. month 3	−65.3	−145.6; 15.1	1.87	0.098
baseline vs. month 6	−56.9	−128.1; 14.2	1.85	0.102
baseline vs. month 12	−62.5	−131.8; 6.8	2.08	0.071
month 3 vs. month 6	8.3	−5.3; 21.9	1.41	0.195
month 3 vs. month 12	2.8	−23.0; 17.5	0.32	0.760
month 6 vs. month 12	5.5	−7.3; 18.4	1.00	0.347

**Table 8:** Motor and non-motor symptoms in increased and combined LEDD groups at baseline.

Symptom	Me (IQR)		Z	*p*
	Increased LEDD	Combined LEDD		
Motor symptoms				
Tremor- + hypokinesia- + rigidity-treated side	14.25 (17.5; 21.5)	12.0 (10.0; 18.5)	−1.4597	0.1444
Tremor-treated side	25.25 (32.0; 48.75)	30.0 (20.5; 39.25)	−0.5789	0.5627
UPDRS	27.75 (37.5; 41.0)	27.0 (18.0; 55.0)	−0.7467	0.4553
Hemi-CRST-untreated side	5.5 (2.0; 9.5)	7.0 (3.5; 12.0)	−0.9638	0.3351
Hemi-CRST-treated side	20.0 (14.0; 23.0)	16.0 (13.0; 25.5)	−0.6417	0.5211
Non-motor symptoms				
ADL	80.0 (72.5; 85.25)	80.0 (75.0; 95.0)	−1.1168	0.2641
Anxiety	6.5 (4.25; 14.5)	5.0 (3.5; 10.5)	−1.1468	0.2515
Apathy	8.0 (6.25; 10.75)	9.0 (8.0; 14.0)	−0.8609	0.3893
Depression	12.0 (6.25; 16.75)	7.0 (2.5; 14.0)	−1.0332	0.3015
LEDD	310.0 (152.5; 806.25)	550.0 (325.0; 862.5)	−0.7845	0.4327
MoCA	25.5 (24.25; 29.25)	24.0 (23.0; 27.5)	−1.4674	0.1423
NMSS	10.5 (5.0; 23.5)	7.0 (2.5; 20.0)	−1.1051	0.2691
QoL	44.5 (19.5; 62.5)	43.0 (15.5; 53.5)	−0.7825	0.4339

**Table 9:** Motor and non-motor symptoms in increased and combined LEDD groups at the end of follow-up.

Symptom	Me (IQR)		Z	*p*
	Increased LEDD	Combined LEDD		
Motor symptoms				
Tremor- + hypokinesia- + rigidity-treated side	11.5 (6.75; 14.0)	6.0 (4.0; 7.5)	−2.4630	0.0138
Tremor-treated side	19.5 (7.25; 23.0)	11.0 (6.0; 15.0)	−1.1030	0.2700
UPDRS	30.5 (22.75; 36.75)	25.0 (16.5; 27.0)	−1.8175	0.0691
Hemi-CRST-untreated side	6.5 (2.5; 9.0)	8.0 (4.5; 12.0)	0.8561	0.391
Hemi-CRST-treated side	11.5 (5.75; 15.0)	3.0 (0.00; 10.0)	−2.3225	0.0202
Non-motor symptoms				
ADL	85.0 (70.0; 97.5)	91.5 (88.0; 96.0)	−0.7720	0.4401
Anxiety	10.5 (7.25; 18.75)	10.0 (4.5; 23.5)	−0.0711	0.9433
Apathy	9.0 (7.25; 16.0)	12.0 (6.0; 16.0)	−0.0357	0.9715
Depression	12.0 (10.0; 16.0)	9.0 (5.0; 15.0)	−1.0763	0.2818
LEDD	551.25 (431.25; 956.25)	500.0 (275.0; 750.0)	−1.3525	0.1762
MoCA	26.5 (24.25; 28.75)	28.0 (25.0; 29.5)	−0.8621	0.3886
NMSS	15.0 (9.0; 22.0)	9.0 (7.0; 22.5)	−0.7139	0.4753
QoL	36.5 (14.0; 54.75)	16.0 (12.0; 39.0)	−0.9600	0.3370

**Table 10:** Motor and non-motor symptoms determining LEDD increase at the end of follow-up (only significant results are shown).

Variable	B	95% CI	T	*p*
progression of tremor + rigidity + hypokinesia between 2nd day and end of the 6th month (treated side)	0.073	0.024; 0.122	3.25	0.0070

**Table 11:** Thalamotomy-related adverse events.

Adverse Event	№ (%) of Patients			
	2nd Day	3 Months	6 Months	12 Months
Hemi- or monoparesis	5 (23.8)	4 (19.0)	3 (14.3)	1 (4.8)
Dysarthria	4 (19.0)	2 (9.5)	1 (4.8)	1 (4.8)
Hemiataxia	4 (19.0)	3 (14.3)	1 (4.8)	1 (4.8)
Parasthesia/numbness orofacial	4 (19.0)	2 (9.5)	2 (9.5)	2 (9.5)
Parasthesia/numbness finger	1 (4.8)	-	-	-

**Table 12:** Changes in nucleus, edema, and FA during follow-up.

Before MRgFUS	3 hrs Post-MRgFUS	Day 2 Post-MRgFUS	3 Months Post-MRgFUS	6 Months Post-MRgFUS	12 Months Post-MRgFUS
NUCLEUS					
-	194 [157; 238]	394 [314; 504.5]	29 [12; 52]	9 [2; 20]	9.5 [4; 24.75]
EDEMA					
-	841 [709–1101]	2718.5 [1974.5; 3426.25]	37 [13.5–50.0]	18 [0.5; 44]	8 [0; 16]
FRACTIONAL ANISOTROPY					
0.39 [0.36; 0.45]	0.15 [0.13; 0.17] z = −4.014; *p* = 0.0001 *	0.16 [0.14; 0.19] z = −3.823; *p* = 0.0001 *	0.31 [0.25; 0.34] z = −2.897; *p* = 0.0038 *	0.32 [0.26; 0.36] z = −1.334; *p* = 0.182 *	0.32 [0.27; 0.57] z = −0.405; *p* = 0.687 *

*—compared with FA before MRgFUS.

## Data Availability

The raw data supporting the conclusions of this article will be made available by the authors on request.

## Abbreviations

The following abbreviations are used in this manuscript:

PD	Parkinson’s disease
ET	Essential tremor
TDPD	Tremor-dominant Parkinson disease
MRgFUS	Magnetic resonance guided focused ultrasound
FUS	Focused ultrasound
MRI	Magnetic resonance imaging
DBS	Deep brain stimulation
AE	Adverse events
SDR	Scull density ratio
DDS	Dopamine dysregulation syndrome
BBB	Blood–brain barrier
LEDD	Levodopa equivalent daily dose
FA	Fractional anisotropy
VL	Ventral lateral nucleus
VIM	Ventral intermediate nucleus
VOP	Ventral oral posterior nucleus
VOA	Ventral oral anterior nucleus
GPi	Globus pallidus internus
MDC	Minimal detectable change
MCID	Minimal clinically important difference
S&E ADL	Schwab and England ADL Scale
ADL	Activities of daily living
PDQ-39	Parkinson’s Disease Quality of Life Questionnaire-39
UPDRS	Unified Parkinson’s Disease Rating Scale
NMSS	Non-Motor Symptoms Scale for Parkinson’s Disease
QUIP	Questionnaire for Impulsive–compulsive Disorders in Parkinson’s Disease Rating Scale
CRST	Clinical Rating Scale for Tremor
MoCA	Montreal Cognitive Assessment
QoL	Quality of life

## References

[1] Thenganatt M.A., Jankovic J. (2014). Parkinson disease subtypes. JAMA Neurol..

[2] Stebbins G.T., Goetz C.G., Burn D.J., Jankovic J., Khoo T.K., Tilley B.C. (2013). How to identify tremor dominant and postural instability/gait difficulty groups with the movement disorder society unified Parkinson’s disease rating scale: Comparison with the unified Parkinson’s disease rating scale. Mov. Disord..

[3] Jankovic J., McDermott M., Carter J., Gauthier S., Goetz C., Golbe L., Huber S., Koller W., Olanow C., Shoulson I. (1990). Variable expression of Parkinson’s disease: A base-line analysis of the DATATOP cohort. The Parkinson Study Group. Neurology.

[4] Zaidel A., Arkadir D., Israel Z., Bergman H. (2009). Akineto-rigid vs. tremor syndromes in *Parkinsonism*. Curr. Opin. Neurol..

[5] Aleksovski D., Miljkovic D., Bravi D., Antonini A. (2018). Disease progression in Parkinson subtypes: The PPMI dataset. Neurol. Sci..

[6] Zach H., Dirkx M.F., Roth D., Pasman J.W., Bloem B.R., Helmich R.C. (2020). Dopamine-responsive and dopamine-resistant resting tremor in Parkinson disease. Neurology.

[7] Pringsheim T., Day G.S., Smith D.B., Rae-Grant A., Licking N., Armstrong M.J., de Bie R.M.A., Roze E., Miyasaki J.M., Hauser R.A. (2021). Guideline Subcommittee of the AAN. Dopaminergic Therapy for Motor Symptoms in Early Parkinson Disease Practice Guideline Summary: A Report of the AAN Guideline Subcommittee. Neurology.

[8] Koller W., Pahwa R., Busenbark K., Hubble J., Wilkinson S., Lang A., Tuite P., Sime E., Lazano A., Hauser R. (1997). High-frequency unilateral thalamic stimulation in the treatment of essential and parkinsonian tremor. Ann. Neurol..

[9] Zirh A., Reich S.G., Dougherty P.M., Lenz F.A. (1999). Stereotactic thalamotomy in the treatment of essential tremor of the upper extremity: Reassessment including a blinded measure of outcome. J. Neurol. Neurosurg. Psychiatry.

[10] Elias W.J., Lipsman N., Ondo W.G., Ghanouni P., Kim Y.G., Lee W., Schwartz M., Hynynen K., Lozano A.M., Shah B.B. (2016). A Randomized Trial of Focused Ultrasound Thalamotomy for Essential Tremor. N. Engl. J. Med..

[11] Jung N.Y., Park C.K., Chang W.S., Jung H.H., Chang J.W. (2018). Effects on cognition and quality of life with unilateral magnetic resonance-guided focused ultrasound thalamotomy for essential tremor. Neurosurg. Focus.

[12] Halpern C.H., Santini V., Lipsman N., Lozano A.M., Schwartz M.L., Shah B.B., Elias W.J., Cosgrove G.R., Hayes M.T., McDannold N. (2019). Three-year follow-up of prospective trial of focused ultrasound thalamotomy for essential tremor. Neurology.

[13] Ito H., Fukutake S., Yamamoto K., Yamaguchi T., Taira T., Kamei T. (2018). Magnetic Resonance Imaging-guided Focused Ultrasound Thalamotomy for Parkinson’s Disease. Intern. Med..

[14] Martínez-Fernández R., Rodríguez-Rojas R., Del Álamo M., Hernández-Fernández F., Pineda-Pardo J.A., Dileone M., Alonso-Frech F., Foffani G., Obeso I., Gasca-Salas C. (2018). Focused ultrasound subthalamotomy in patients with asymmetric Parkinson’s disease: A pilot study. Lancet Neurol..

[15] Sinai A., Nassar M., Sprecher E., Constantinescu M., Zaaroor M., Schlesinger I. (2022). Focused Ultrasound Thalamotomy in Tremor Dominant Parkinson’s Disease: Long-Term Results. J. Park. Dis..

[16] Krishna V., Fishman P.S., Eisenberg H.M., Kaplitt M., Baltuch G., Chang J.W., Chang W.C., Martinez Fernandez R., Del Alamo M., Halpern C.H. (2023). Trial of Globus Pallidus Focused Ultrasound Ablation in Parkinson’s Disease. N. Engl. J. Med..

[17] Jameel A., Akgun S., Yousif N., Smith J., Jones B., Nandi D., Bain P., Gedroyc W. (2024). The evolution of ventral intermediate nucleus targeting in MRI-guided focused ultrasound thalamotomy for essential tremor: An international multi-center evaluation. Front. Neurol..

[18] Segar D.J., Lak A.M., Lee S., Harary M., Chavakula V., Lauro P., McDannold N., White J., Cosgrove G.R. (2021). Lesion location and lesion creation affect outcomes after focused ultrasound thalamotomy. Brain.

[19] Bond A.E., Shah B.B., Huss D.S., Dallapiazza R.F., Warren A., Harrison M.B., Sperling S.A., Wang X.Q., Gwinn R., Witt J. (2017). Safety and Efficacy of Focused Ultrasound Thalamotomy for Patients with Medication-Refractory, Tremor-Dominant Parkinson Disease: A Randomized Clinical Trial. JAMA Neurol..

[20] Sperling S.A., Shah B.B., Barrett M.J., Bond A.E., Huss D.S., Gonzalez Mejia J.A., Elias W.J. (2018). Focused ultrasound thalamotomy in Parkinson disease: Nonmotor outcomes and quality of life. Neurology.

[21] Yamamoto K., Ito H., Fukutake S., Odo T., Kamei T., Yamaguchi T., Taira T. (2021). Focused Ultrasound Thalamotomy for Tremor-dominant Parkinson’s Disease: A Prospective 1-year Follow-up Study. Neurol. Med. Chir..

[22] Saporito G., Sucapane P., Ornello R., Cerone D., Bruno F., Splendiani A., Masciocchi C., Ricci A., Marini C., Sacco S. (2023). Cognitive outcomes after focused ultrasound thalamotomy for tremor: Results from the COGNIFUS (COGNitive in Focused UltraSound) study. Park. Relat. Disord..

[23] Postuma R.B., Berg D., Stern M., Poewe W., Olanow C.W., Oertel W., Obeso J., Marek K., Litvan I., Lang A.E. (2015). MDS clinical diagnostic criteria for Parkinson’s disease. Mov. Disord..

[24] Fahn S., Tolosa E., Marin C., Jankovic J., Tolosa E. (1988). Clinical rating scale for tremor. Parkinson’s Disease and Movement Disorder.

[25] Fahn S., Elton R.L., Fahn S., Marsden C.D., Goldstein M., Calne D.B., UPDRS program members (1987). Unified Parkinsons Disease Rating Scale. Recent Developments in Parkinsons Disease.

[26] Tinetti M.E. (1986). Performance-oriented assessment of mobility problems in elderly patients. J. Am. Geriatr. Soc..

[27] Chaudhuri K.R., Martinez-Martin P. (2008). Quantitation of non-motor symptoms in Parkinson’s disease. Eur. J. Neurol..

[28] Nasreddine Z.S., Phillips N.A., Bédirian V., Charbonneau S., Whitehead V., Collin I., Cummings J.L., Chertkow H. (2005). The Montreal Cognitive Assessment, MoCA: A brief screening tool for mild cognitive impairment. J. Am. Geriatr. Soc..

[29] Beck A.T., Ward C.H., Mendelson M., Mock J., Erbaugh J. (1961). An inventory for measuring depression. Arch. Gen. Psychiatry.

[30] Beck A.T., Epstein N., Brown G., Steer R.A. (1988). An inventory for measuring clinical anxiety: Psychometric properties. J. Consult. Clin. Psychol..

[31] Starkstein S.E., Mayberg H.S., Preziosi T.J., Andrezejewski P., Leiguarda R., Robinson R.G. (1992). Reliability, validity, and clinical correlates of apathy in Parkinson’s disease. J. Neuropsychiatry Clin. Neurosci..

[32] Weintraub D., Hoops S., Shea J.A., Lyons K.E., Pahwa R., Driver-Dunckley E.D., Adler C.H., Potenza M.N., Miyasaki J., Siderowf A.D. (2009). Validation of the questionnaire for impulsive-compulsive disorders in Parkinson’s disease. Mov. Disord..

[33] Schwab R., England A., Gillingham J.F., Donaldson I.M.L. (1969). Projection technique for evaluating surgery in Parkinson’s disease. Third Symposium on Parkinson’s Disease.

[34] Peto V., Jenkinson C., Fitzpatrick R. (1998). PDQ-39: A review of the development, validation and application of a Parkinson’s disease quality of life questionnaire and its associated measures. J. Neurol..

[35] Tomlinson C.L., Stowe R., Patel S., Rick C., Gray R., Clarke C.E. (2010). Systematic review of levodopa dose equivalency reporting in Parkinson’s disease. Mov. Disord..

[36] Clavien P.A., Barkun J., de Oliveira M.L., Vauthey J.N., Dindo D., Schulick R.D., de Santibañes E., Pekolj J., Slankamenac K., Bassi C. (2009). The Clavien-Dindo classification of surgical complications: Five-year experience. Ann. Surg..

[37] Gumin I.S., Malykhina E.A., Dzhafarov V.M., Katunina E.A., Senko I.V., Dolgushin M.B. (2022). First experience of thalamotomy by focused ultrasound under MR-guided navigation in the treatment of tremor. Neuroimaging follow-up. Case report and literature review. Burdenko’s J. Neurosurg..

[38] Benabid A.L., Pollak P., Gervason C., Hoffmann D., Gao D.M., Hommel M., Perret J.E., de Rougemont J. (1991). Long-term suppression of tremor by chronic stimulation of the ventral intermediate thalamic nucleus. Lancet.

[39] Zaaroor M., Sinai A., Goldsher D., Eran A., Nassar M., Schlesinger I. (2018). Magnetic resonance-guided focused ultrasound thalamotomy for tremor: A report of 30 Parkinson’s disease and essential tremor cases. J. Neurosurg..

[40] Dolgushin M.B., Prishchepina K.A., Martynov M.Y., Gumin I.S., Katunina E.A., Senko I.V., Tairova R.T., Dvoryanchikov A.V. (2025). Dynamics of permeability of the blood-brain barrier after FUS thalamotomy according to contrast-enhanced MRI. Burdenko’s J. Neurosurg..

[41] Wu C.Y., Hung S.J., Lin K.C., Chen K.H., Chen P., Tsay P.K. (2019). Responsiveness, Minimal Clinically Important Difference, and Validity of the MoCA in Stroke Rehabilitation. Occup. Ther. Int..

[42] Lindvall E., Abzhandadze T., Quinn T.J., Sunnerhagen K.S., Lundström E. (2024). Is the difference real, is the difference relevant: The minimal detectable and clinically important changes in the Montreal Cognitive Assessment. Cereb. Circ. Cogn. Behav..

[43] Lin F., Wu D., Yu J., Weng H., Chen L., Meng F., Chen Y., Ye Q., Cai G. (2021). Comparison of efficacy of deep brain stimulation and focused ultrasound in parkinsonian tremor: A systematic review and network meta-analysis. J. Neurol. Neurosurg. Psychiatry.

[44] Liang M., Hou L., Liang J., Bao S. (2025). Ameliorating motor performance and quality of life in Parkinson’s disease: A comparison of deep brain stimulation and focused ultrasound surgery. Front. Neurol..

[45] Cesarano S., Saporito G., Sucapane P., Bruno F., Catalucci A., Pistoia M.L., Splendiani A., Ricci A., Di Cesare E., Totaro R. (2024). Staged magnetic resonance-guided focused ultrasound thalamotomy for the treatment of bilateral essential tremor and Parkinson’s disease related tremor: A systematic review and critical appraisal of current knowledge. Front. Neurol..

[46] Martínez-Fernández R., Natera-Villalba E., Rodríguez-Rojas R., Del Álamo M., Pineda-Pardo J.A., Obeso I., Guida P., Jiménez-Castellanos T., Pérez-Bueno D., Duque A. (2024). Staged Bilateral MRI-Guided Focused Ultrasound Subthalamotomy for Parkinson Disease. JAMA Neurol..

[47] Bruno F., Catalucci A., Arrigoni F., Gagliardi A., Campanozzi E., Corridore A., Tommasino E., Pagliei V., Pertici L., Palumbo P. (2021). Comprehensive Evaluation of Factors Affecting Tremor Relapse after MRgFUS Thalamotomy: A Case-Control Study. Brain Sci..

[48] Braccia A., Golfrè Andreasi N., Ghielmetti F., Aquino D., Savoldi A.P., Cilia R., Telese R., Colucci F., Gaudiano G., Romito L.M. (2025). Magnetic Resonance-Guided Focused Ultrasound Thalamotomy in a Prospective Cohort of 52 Patients with Parkinson’s Disease: A Possible Critical Role of Age and Lesion Volume for Predicting Tremor Relapse. Mov. Disord..

[49] Jang C., Park H.J., Chang W.S., Pae C., Chang J.W. (2016). Immediate and Longitudinal Alterations of Functional Networks after Thalamotomy in Essential Tremor. Front. Neurol..

[50] Helmich R.C., Janssen M.J., Oyen W.J., Bloem B.R., Toni I. (2011). Pallidal dysfunction drives a cerebellothalamic circuit into Parkinson tremor. Ann. Neurol..

[51] Qamhawi Z., Towey D., Shah B., Pagano G., Seibyl J., Marek K., Borghammer P., Brooks D.J., Pavese N. (2015). Clinical correlates of raphe serotonergic dysfunction in early Parkinson’s disease. Brain.

[52] Underwood C.F., Parr-Brownlie L.C. (2021). Primary motor cortex in Parkinson’s disease: Functional changes and opportunities for neurostimulation. Neurobiol. Dis..

[53] Golfrè Andreasi N., Cilia R., Romito L.M., Bonvegna S., Straccia G., Elia A.E., Novelli A., Messina G., Tringali G., Levi V. (2022). Magnetic Resonance-Guided Focused Ultrasound Thalamotomy May Spare Dopaminergic Therapy in Early-Stage Tremor-Dominant Parkinson’s Disease: A Pilot Study. Mov. Disord..

[54] Chua M.M.J., Blitz S.E., Ng P.R., Segar D.J., McDannold N.J., White P.J., Christie S., Hayes M.T., Rolston J.D., Cosgrove G.R. (2023). Focused Ultrasound Thalamotomy for Tremor in Parkinson’s Disease: Outcomes in a Large, Prospective Cohort. Mov. Disord..

[55] Purrer V., Pohl E., Borger V., Weiland H., Boecker H., Schmeel F.C., Wüllner U. (2024). Motor and non-motor outcome in tremor dominant Parkinson’s disease after MR-guided focused ultrasound thalamotomy. J. Neurol..

[56] Sammartino F., Krishna V., King N.K., Lozano A.M., Schwartz M.L., Huang Y., Hodaie M. (2016). Tractography-Based Ventral Intermediate Nucleus Targeting: Novel Methodology and Intraoperative Validation. Mov. Disord..

[57] Miller T.R., Zhuo J., Eisenberg H.M., Fishman P.S., Melhem E.R., Gullapalli R., Gandhi D. (2019). Targeting of the dentato-rubro-thalamic tract for MR-guided focused ultrasound treatment of essential tremor. Neuroradiol. J..

[58] Hassler R., Reichert T. (1954). Indikationen und Lokalisationsmethode der gezielten Hirnoperationen [Indications and localization of stereotactic brain operations]. Nervenarzt.

[59] Macchi G., Jones E.G. (1997). Toward an agreement on terminology of nuclear and subnuclear divisions of the motor thalamus. J. Neurosurg..

[60] Owen R.L., Grewal S.S., Thompson J.M., Hassan A., Lee K.H., Klassen B.T. (2022). Effectiveness of Thalamic Ventralis Oralis Anterior and Posterior Nuclei Deep Brain Stimulation for Posttraumatic Dystonia. Mayo Clin. Proc. Innov. Qual. Outcomes.

[61] Hyam J.A., Owen S.L., Kringelbach M.L., Jenkinson N., Stein J.F., Green A.L., Aziz T.Z. (2012). Contrasting connectivity of the ventralis intermedius and ventralis oralis posterior nuclei of the motor thalamus demonstrated by probabilistic tractography. Neurosurgery.

[62] Parras O., Domínguez P., Tomás-Biosca A., Guridi J. (2022). The role of tractography in the localisation of the Vim nucleus of the thalamus and the dentatorubrothalamic tract for the treatment of tremor. Neurologia.

[63] Ohye C., Shibazaki T., Hirato M., Kawashima Y., Matsumura M. (1990). Strategy of selective VIM thalamotomy guided by microrecording. Stereotact. Funct. Neurosurg..

[64] Martínez-Fernández R., Máñez-Miró J.U., Rodríguez-Rojas R., Del Álamo M., Shah B.B., Hernández-Fernández F., Pineda-Pardo J., Monje M.H.G., Fernández-Rodríguez B., Sperling S.A. (2020). Randomized Trial of Focused Ultrasound Subthalamotomy for Parkinson’s Disease. N. Engl. J. Med..

[65] Ito H., Yamamoto K., Fukutake S., Kamei T., Yamaguchi T., Taira T. (2021). Two-year follow-up results of magnetic resonance imaging-guided focused ultrasound unilateral pallidotomy for Parkinson’s disease. Neurol. Clin. Neurosci..

[66] Horváth K., Aschermann Z., Kovács M., Makkos A., Harmat M., Janszky J., Komoly S., Karádi K., Kovács N. (2017). Changes in Quality of Life in Parkinson’s Disease: How Large Must They Be to Be Relevant?. Neuroepidemiology.

[67] Monteiro J.D.S., Silva B.B.E., de Oliveira R.R., Borges P.G.L.B., Pereira M.A.O.M., Costa K.A., Nunes A.L.S., Telles J.P.M., Valença M.M. (2024). Magnetic resonance-guided focused ultrasound ventral intermediate thalamotomy for Tremor-Dominant Parkinson’s disease: A systematic review and meta-analysis. Neurosurg. Rev..

[68] Shi Y., Dobkin R., Weintraub D., Cho H.R., Caspell-Garcia C., Bock M., Brown E., Aarsland D., Dahodwala N. (2024). Association of Baseline Depression and Anxiety with Longitudinal Health Outcomes in Parkinson’s Disease. Mov. Disord. Clin. Pract..

[69] Bouça-Machado R., Fernandes A., Ranzato C., Beneby D., Nzwalo H., Ferreira J.J. (2022). Measurement tools to assess activities of daily living in patients with Parkinson’s disease: A systematic review. Front. Neurosci..

[70] Hariz G.M., Forsgren L. (2011). Activities of daily living and quality of life in persons with newly diagnosed Parkinson’s disease according to subtype of disease, and in comparison to healthy controls. Acta Neurol. Scand..

[71] Saporito G., Sucapane P., Bruno F., Catalucci A., Masciocchi C., Pistoia M.L., Splendiani A., Ricci A., Di Cesare E., Marini C. (2024). Cognitive safety of focused ultrasound thalamotomy for tremor: 1-year follow-up results of the COGNIFUS part 2 study. Front. Neurol..

[72] De Micco R., Satolli S., Siciliano M., Di Nardo F., Caiazzo G., Russo A., Giordano A., Esposito F., Tedeschi G., Tessitore A. (2021). Connectivity Correlates of Anxiety Symptoms in Drug-Naive Parkinson’s Disease Patients. Mov. Disord..

[73] Boccalini C., Carli G., Pilotto A., Padovani A., Perani D. (2022). Gender differences in dopaminergic system dysfunction in de novo Parkinson’s disease clinical subtypes. Neurobiol. Dis..

[74] Carey G., Viard R., Lopes R., Kuchcinski G., Defebvre L., Leentjens A.F., Dujardin K. (2023). Anxiety in Parkinson’s Disease Is Associated with Changes in Brain Structural Connectivity. J. Park. Dis..

[75] Dan R., Růžička F., Bezdicek O., Růžička E., Roth J., Vymazal J., Goelman G., Jech R. (2017). Separate neural representations of depression, anxiety and apathy in Parkinson’s disease. Sci. Rep..

[76] Yamanishi T., Tachibana H., Oguru M., Matsui K., Toda K., Okuda B., Oka N. (2013). Anxiety and depression in patients with Parkinson’s disease. Intern. Med..

[77] Lin H., Cai X., Zhang D., Liu J., Na P., Li W. (2020). Functional connectivity markers of depression in advanced Parkinson’s disease. Neuroimage Clin..

[78] Rohringer C.R., Sewell I.J., Gandhi S., Isen J., Davidson B., McSweeney M., Swardfager W., Scantlebury N., Swartz R.H., Hamani C. (2022). Cognitive effects of unilateral thalamotomy for tremor: A meta-analysis. Brain Commun..

[79] Petersen J., McGough J., Gopinath G., Scantlebury N., Tripathi R., Brandmeir C., Boshmaf S.Z., Brandmeir N.J., Sewell I.J., Konrad P.E. (2024). Cognitive outcomes following unilateral magnetic resonance-guided focused ultrasound thalamotomy for essential tremor: Findings from two cohorts. Brain Commun..

[80] Wang Y. (2024). Thalamus and its functional connections with cortical regions contribute to complexity-dependent cognitive reasoning. Neuroscience.

[81] Wen Z., Zhang J., Li J., Dai J., Lin F., Wu G. (2016). Altered Activation in Cerebellum Contralateral to Unilateral Thalamotomy May Mediate Tremor Suppression in Parkinson’s Disease: A Short-Term Regional Homogeneity fMRI Study. PLoS ONE.

[82] Dahmani L., Bai Y., Li M., Ren J., Shen L., Ma J., Li H., Wei W., Li P., Wang D. (2023). Focused ultrasound thalamotomy for tremor treatment impacts the cerebello-thalamo-cortical network. npj Park. Dis..

[83] Fiorenzato E., Biundo R., Cecchin D., Frigo A.C., Kim J., Weis L., Strafella A.P., Antonini A. (2018). Brain Amyloid Contribution to Cognitive Dysfunction in Early-Stage Parkinson’s Disease: The PPMI Dataset. J. Alzheimers Dis..

[84] Park S.H., Baik K., Jeon S., Chang W.S., Ye B.S., Chang J.W. (2021). Extensive frontal focused ultrasound mediated blood-brain barrier opening for the treatment of Alzheimer’s disease: A proof-of-concept study. Transl. Neurodegener..

[85] Rezai A.R., D’Haese P.F., Finomore V., Carpenter J., Ranjan M., Wilhelmsen K., Mehta R.I., Wang P., Najib U., Vieira Ligo Teixeira C. (2024). Ultrasound Blood-Brain Barrier Opening and Aducanumab in Alzheimer’s Disease. N. Engl. J. Med..

[86] Gasca-Salas C., Fernández-Rodríguez B., Pineda-Pardo J.A., Rodríguez-Rojas R., Obeso I., Hernández-Fernández F., Del Álamo M., Mata D., Guida P., Ordás-Bandera C. (2021). Blood-brain barrier opening with focused ultrasound in Parkinson’s disease dementia. Nat. Commun..

[87] Pineda-Pardo J.A., Gasca-Salas C., Fernández-Rodríguez B., Rodríguez-Rojas R., Del Álamo M., Obeso I., Hernández-Fernández F., Trompeta C., Martínez-Fernández R., Matarazzo M. (2022). Striatal Blood-Brain Barrier Opening in Parkinson’s Disease Dementia: A Pilot Exploratory Study. Mov. Disord..

[88] Jeong H., Im J.J., Park J.S., Na S.H., Lee W., Yoo S.S., Song I.U., Chung Y.A. (2021). A pilot clinical study of low-intensity transcranial focused ultrasound in Alzheimer’s disease. Ultrasonography.

[89] Khan A., Ezeugwa J., Ezeugwu V.E. (2024). A systematic review of the associations between sedentary behavior, physical inactivity, and non-motor symptoms of Parkinson’s disease. PLoS ONE.

[90] Kim R., Lee T.L., Lee H., Ko D.K., Lee J.H., Shin H., Lim D., Jun J.S., Byun K., Park K. (2023). Effects of physical exercise interventions on cognitive function in Parkinson’s disease: An updated systematic review and meta-analysis of randomized controlled trials. Park. Relat. Disord..

[91] Diaz-Galvan P., Franco-Rosado P., Silva-Rodriguez J., Castro-Labrador S., Labrador-Espinosa M.A., Muñoz-Delgado L., Grothe M.J., Mir P. (2025). Association of Physical Exercise with Structural Brain Changes and Cognitive Decline in Patients with Early Parkinson Disease. Neurology.

[92] Walsh E.I., Smith L., Northey J., Rattray B., Cherbuin N. (2020). Towards an understanding of the physical activity-BDNF-cognition triumvirate: A review of associations and dosage. Ageing Res. Rev..

[93] Leem Y.H., Park J.S., Park J.E., Kim D.Y., Kim H.S. (2023). Suppression of neuroinflammation and α-synuclein oligomerization by rotarod walking exercise in subacute MPTP model of Parkinson’s disease. Neurochem. Int..

[94] Maass A., Düzel S., Goerke M., Becke A., Sobieray U., Neumann K., Lövden M., Lindenberger U., Bäckman L., Braun-Dullaeus R. (2015). Vascular hippocampal plasticity after aerobic exercise in older adults. Mol. Psychiatry.

[95] Lin L., He Y.X., Wen Q., Liu J.Y., Dai Y., Fei Y.Z., Li H., Li C.Q., Zhou H. (2025). Evaluation of the efficacy of Tai Chi on the cognitive function of patients with mild cognitive dysfunction and research on its mechanism. Front. Aging Neurosci..

[96] Kim Y.J., Han K.D., Baek M.S., Cho H., Lee E.J., Lyoo C.H. (2020). Association between physical activity and conversion from mild cognitive impairment to dementia. Alzheimers Res. Ther..

[97] Demurtas J., Schoene D., Torbahn G., Marengoni A., Grande G., Zou L., Petrovic M., Maggi S., Cesari M., Lamb S. (2020). Physical Activity and Exercise in Mild Cognitive Impairment and Dementia: An Umbrella Review of Intervention and Observational Studies. J. Am. Med. Dir. Assoc..

[98] Zur G., Lesman-Segev O.H., Schlesinger I., Goldsher D., Sinai A., Zaaroor M., Assaf Y., Eran A., Kahn I. (2020). Tremor Relief and Structural Integrity after MRI-guided Focused US Thalamotomy in Tremor Disorders. Radiology.

[99] Boecker H., Schild H.H., Kindler C., Schmitt A., Solymosi L., Wüllner U., Pieper C.C. (2020). MRI follow-up after magnetic resonance-guided focused ultrasound for non-invasive thalamotomy: The neuroradiologist’s perspective. Neuroradiology.

[100] Alexander A.L., Lee J.E., Lazar M., Field A.S. (2007). Diffusion tensor imaging of the brain. Neurotherapeutics.

[101] Gumin I.S., Nikitin D.V., Shipilova N.N., Katunina E.A., Senko I.V., Dolgushin M.B. (2024). Fractional anisotropy within zone of destruction, tremor evaluation and MRI manifestation follow up after focused ultrasound thalamotomy for patients with Parkinson’s disease. Med. Vis..

[102] França C., Cury R.G. (2025). The best of both worlds: Deep brain stimulation or high-frequency focused ultrasound for tremor refractory syndromes. Arq. Neuropsiquiatr..

[103] Thomas B., Bellini G., Lee W.Y., Shi Y., Mogilner A., Pourfar M.H. (2025). High Intensity Focused Ultrasound—Longitudinal Data on Efficacy and Safety. Tremor Other Hyperkinetic Mov..

[104] Massruhá K.S., Cardoso E.F. (2025). High-intensity focused ultrasound (HIFU) versus deep brain stimulation (DBS) for refractory tremor: Team HIFU. Arq. Neuropsiquiatr..

